# Community pharmacy-delivered interventions for public health priorities: a systematic review of interventions for alcohol reduction, smoking cessation and weight management, including meta-analysis for smoking cessation

**DOI:** 10.1136/bmjopen-2015-009828

**Published:** 2016-02-29

**Authors:** Tamara J Brown, Adam Todd, Claire O'Malley, Helen J Moore, Andrew K Husband, Clare Bambra, Adetayo Kasim, Falko F Sniehotta, Liz Steed, Sarah Smith, Lucie Nield, Carolyn D Summerbell

**Affiliations:** 1 School of Medicine, Pharmacy and Health, Durham University Queen's Campus, Stockton-on-Tees, UK; 2 FUSE, UKCRC Centre for Translational Research in Public Health, Newcastle University, Newcastle Upon Tyne, UK; 3 Wolfson Research Institute for Health and Wellbeing, Durham University Queen's Campus, Stockton-on-Tees, UK; 4 Centre for Health and Inequalities Research, Department of Geography, Durham University, Durham, UK; 5 Institute of Health & Society, Newcastle University, Newcastle Upon Tyne, UK; 6 Blizard Institute, Barts and The London School of Medicine and Dentistry, London, UK; 7 Sheffield Business School, Sheffield Hallam University, Sheffield, UK

**Keywords:** NUTRITION & DIETETICS, PREVENTIVE MEDICINE, PUBLIC HEALTH

## Abstract

**Objectives:**

To systematically review the effectiveness of community pharmacy-delivered interventions for alcohol reduction, smoking cessation and weight management.

**Design:**

Systematic review and meta-analyses. 10 electronic databases were searched from inception to May 2014.

**Eligibility criteria for selecting studies:**

*Study design*: randomised and non-randomised controlled trials; controlled before/after studies, interrupted times series. *Intervention*: any relevant intervention set in a community pharmacy, delivered by the pharmacy team. No restrictions on duration, country, age, or language.

**Results:**

19 studies were included: 2 alcohol reduction, 12 smoking cessation and 5 weight management. Study quality rating: 6 ‘strong’, 4 ‘moderate’ and 9 ‘weak’. 8 studies were conducted in the UK, 4 in the USA, 2 in Australia, 1 each in 5 other countries. Evidence from 2 alcohol-reduction interventions was limited. Behavioural support and/or nicotine replacement therapy are effective and cost-effective for smoking cessation: pooled OR was 2.56 (95% CI 1.45 to 4.53) for active intervention vs usual care. Pharmacy-based interventions produced similar weight loss compared with active interventions in other primary care settings; however, weight loss was not sustained longer term in a range of primary care and commercial settings compared with control. Pharmacy-based weight management interventions have similar provider costs to those delivered in other primary care settings, which are greater than those delivered by commercial organisations. Very few studies explored if and how sociodemographic or socioeconomic variables moderated intervention effects. Insufficient information was available to examine relationships between effectiveness and behaviour change strategies, implementation factors, or organisation and delivery of interventions.

**Conclusions:**

Community pharmacy-delivered interventions are effective for smoking cessation, and demonstrate that the pharmacy is a feasible option for weight management interventions. Given the potential reach, effectiveness and associated costs of these interventions, commissioners should consider using community pharmacies to help deliver public health services.

**Strengths and limitations of this study**

- To the best of our knowledge, this is the first systematic review that combines evidence from community pharmacy-delivered alcohol, smoking and weight management interventions, and directly compares these findings with other primary care and community healthcare settings.
- This review provides healthcare commissioners with useful evidence on reach, effectiveness and costs when considering using community pharmacies to help deliver smoking cessation and weight management services.
- There was insufficient evidence to assess the effectiveness of community pharmacy-based interventions on health equity.
- The descriptions available did not allow for the coding of specific aspects of theory and behavioural content of the interventions.
- Insufficient information was available to examine the relationship between intervention effectiveness and behaviour change strategies and/or models used, implementation factors, or the organisation and delivery of interventions.

## Introduction

A number of agencies and countries, including WHO, have set a clear strategy for the future of public health. This agenda is focused on improving the healthy life expectancy of the population and, where possible, reducing or removing threats to this aim.^1^ One strand within this agenda is to create accessible, multidisciplinary networks of public health professionals who work within communities and provide services to address key public health issues, health inequalities, and ultimately improve health and well-being. Worldwide, community pharmacies may be an important component of this agenda; WHO acknowledges that community pharmacies and their staff are easily accessible and, as such, could play a key role in delivering public health initiatives, especially in priority areas.^2^ For example, in England, community pharmacies are more accessible than general practice (GP) services.^3^ A recent study has also demonstrated that, in England, 89% of the population can walk to a community pharmacy within 20 min. Significantly, in areas of highest deprivation, this value increases to almost 100%—the so-called positive pharmacy care law.^4^ Community pharmacies could, therefore, be a way of engaging with hard-to-reach populations.

In view of this, many community pharmacies in some countries, now offer smoking cessation services, and a few offer alcohol and weight reduction services.^5^ These services are delivered by pharmacists, pharmacy technicians and/or medicine counter assistants, with a view to modifying health-related behaviours. The specific types of services are wide ranging and include two main approaches: pharmaceutical-related (eg, supplying nicotine replacement therapy (NRT), monitoring of biochemical markers) and non-pharmaceutical-related (eg, providing advice on behaviour change strategies), or a combination of both approaches. Funding arrangements for these services vary by country; in the UK, at present, many of these services are commissioned by the local authority according to local need, and delivered according to an agreed framework. Currently, six Local Pharmaceutical Committees (LPCs) have weight management services, 14 LPCs have alcohol reduction services, and there are 81 stop smoking services.^6^

In 2008, the Department of Health for England^7^ stated it was important to develop ‘a sound evidence base that demonstrates how pharmacy delivers effective, high quality and value for money services’. Reviews published since 2008 have attempted to summarise this evidence base, but the lack of relevant randomised controlled trials (RCTs) have limited their findings.^8–10^ However, a scoping search performed in 2013 revealed a number of relevant RCTs that had, or were about to report their findings; a number of relevant controlled trials were also identified, that could usefully inform the evidence base where there was a lack of evidence from RCTs. The primary objective of this review, therefore, was to systematically review the effectiveness of community pharmacy-delivered interventions for alcohol reduction, smoking cessation and weight management. The secondary objectives were to explore if and how age, ethnicity, gender and socioeconomic status (SES), moderate intervention effects; and to describe how the interventions have been implemented, organised and delivered.

## Methods

The review was funded by the National Institute for Health Research Public Health Research Programme (project number 12/153/52). The review was carried out using the principles outlined in the Cochrane Handbook for Systematic Reviews of Interventions.^11^ The protocol is published in *BMC Systematic Reviews*,^12^ and is registered with the International Prospective Register of Systematic Reviews (PROSPERO CRD42013005943). A review advisory group comprising patients, pharmacists and researchers, helped to guide the research. The review is reported according to the Preferred Reporting Items for Systematic Reviews and Meta-Analyses (PRISMA) guidelines.^13^ ^14^

### Interventions

The review included any type of community pharmacy-delivered intervention aimed at alcohol reduction, smoking cessation, or weight management; of any duration, based in any country and of any age. The setting of interest was the community pharmacy, which was defined as a pharmacy set in the community, which is accessible to all and not based in a hospital, clinic or online. Where a pharmacy is referred to throughout this paper, we refer to a community pharmacy. There was no restriction on the type of comparator, which could be a non-active control, usual care, or another type of active intervention, set in or out of the community pharmacy. Participants could be recruited from outside of the community pharmacy as long as one of the intervention groups was delivered from the community pharmacy. The intervention had to be delivered by the community pharmacist, pharmacy technician or medicines counter assistant; however, the intervention could also include other deliverers as part of a multidisciplinary team.

### Study design

A broad range of controlled study designs were included, using the Cochrane Effective Practice and Organisation of Care (EPOC) study design criteria.^15^ These included RCTs; non-RCTs (nRCT); controlled before/after studies (CBA); interrupted time series (ITS), and repeated measures studies. We included both fully powered and pilot studies; studies were graded lower on quality if they were insufficiently powered.

### Search strategy

Ten electronic databases were searched: Applied Social Sciences Index and Abstracts, Cumulative Index to Nursing and Allied Health Literature, EMBASE, International Bibliography of the Social Sciences, MEDLINE, NHS Economic Evaluation Database, PsycINFO, Social Science Citation Index, Scopus and the Sociological Abstracts; from inception to May 2014 (see online supplementary file 1). Supplementary searches to identify published, unpublished and ongoing studies included bibliographies, contacting experts, grey literature (OpenGrey, Social Care Online, Prevention Information & Evidence elibrary and Nexus UK), study registers (International Standard Registered Clinical/soCial sTudy Number registry and the National Research Register) and website (Google).

10.1136/bmjopen-2015-009828.supp1 Supplementary data

### Outcomes

Interventions for alcohol reduction and smoking cessation had to report a relevant behavioural outcome, and interventions for weight had to report an anthropometric outcome. These outcomes were considered the primary outcomes, and could be measured or self-reported. Where studies reported if and how sociodemographic (age, ethnicity, gender) and/or SES (education, income, occupation, social class, deprivation or poverty) moderated intervention effects on the primary outcomes, this is reported in the review.

The review also describes how the interventions have been organised, implemented and delivered using the methodological tool for the assessment of the implementation of complex public health interventions in systematic reviews, developed by Egan *et al*^16^ for the workplace, and adapted by Bambra *et al*^17^ for obesity interventions. The Behaviour Change Wheel^18^ and the Nuffield Intervention Ladder^19^ were used to broadly describe the behavioural strategies, intervention functions and policy categories of the interventions.

### Data extraction and quality appraisal

Three reviewers (CO, HM, SS) screened the titles and abstracts and two reviewers (CO, TB) screened the full-text articles. Data extraction and quality assessment were conducted independently by TB and one other reviewer (from among AT, CO, CS, HM, LN, LS, SS). Study quality was appraised using the Effective Public Health Practice Project Quality Assessment Tool for Quantitative Studies,^20^ which is recommended by the Cochrane Public Health Review Group.^21^ Studies were assessed for quality using six criteria: selection bias, study design, confounders, blinding, data collection methods and withdrawals/dropouts. Each study was given an overall (global) rating based on the ratings for the six criteria: ‘strong’ (no ‘weak’ ratings), ‘moderate’ (one ‘weak’ rating) and ‘weak’ (two or more ‘weak’ ratings). Any discrepancies in the data extraction or quality assessment were resolved through discussion, or referred to a third reviewer (CS) for final assessment. Extraction of contextual data was conducted by one reviewer (CS) and checked by another (TB). Assessment of behaviour change strategies used was conducted by one reviewer (CS) and checked by two others (FS and LS).

### Analysis and synthesis

Narrative synthesis was conducted for all the included interventions. Owing to the heterogeneity of the studies, it was only possible to conduct meta-analyses for the smoking cessation studies. The smoking data was analysed (AK) using binomial-normal random effect model (R package meta). In order to explain the observed heterogeneity between studies, four different meta-regression models were fitted, accounting for whether the comparator was an active control or usual care, duration of the intervention and the global quality assessment ratings. Q-statistics and the percentage of heterogeneity between studies were reported for each meta-regression model. The most optimal meta-regression model was chosen using a minimum Akaike Information criterion. Owing to the limited available data and lack of informative priors, subgroup analysis by demographic or SES was not considered. A funnel plot for the smoking cessation RCTs was carried out to indicate the possible presence of publication bias and other biases.

## Results

**Figure 1:**
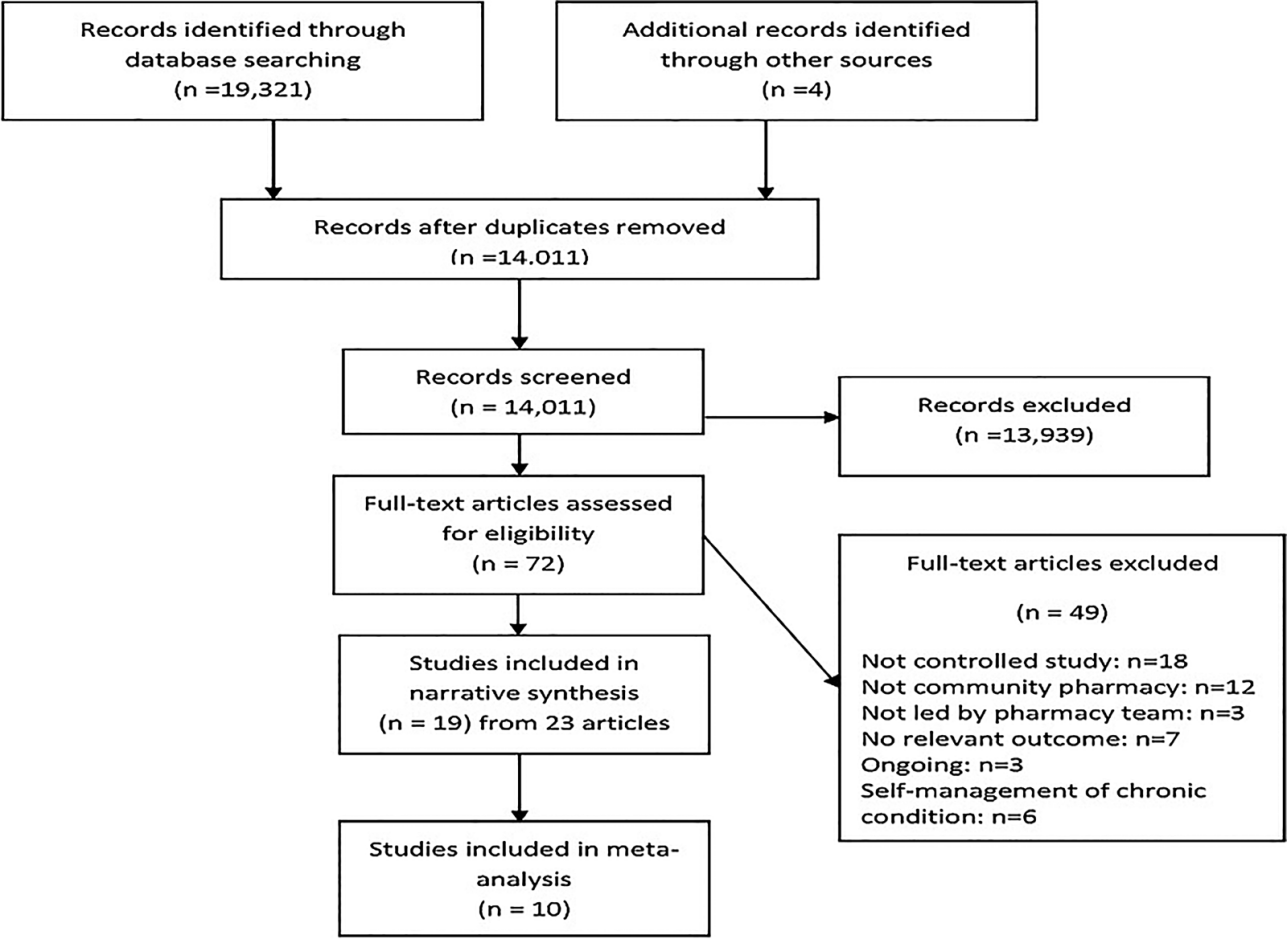
Preferred Reporting Items for Systematic Reviews and Meta-Analyses (PRISMA) flow diagram.

The electronic search identified over 19 000 records, of which 72 full-text articles were screened for eligibility; 19 studies (from 23 articles) were included, and 49 were excluded. Five excluded studies^22–26^ (from six articles) were pharmacotherapy plus lifestyle advice interventions in participants with comorbidities. These studies were excluded because the primary focus was not alcohol, smoking or weight management; these interventions focused on self-management of a chronic condition. The process of inclusion and exclusion of studies are shown in figure 1.

### Study characteristics

Tables 1–3 provide the main study characteristics for all 19 interventions (see online supplementary file 2) for detailed study characteristics, including sociodemographic and SES). There were 2 alcohol reduction interventions,^27^ ^28^ 12 smoking cessation interventions^29–40^ and 5 weight management interventions.^41–45^ There were 15 RCTs, 2 nRCTs^25^ ^33^ ^44^ and 2 CBAs.^29^ ^29^ ^42^ There were 17 published journal articles and two reports.^28^ ^42^ Eight studies were conducted in the UK,^27–29^ ^33^ ^36^ ^38^ ^42^ ^43^ four in the USA,^30^ ^35^ ^41^ ^44^ two in Australia^31^ ^39^ and one each in Canada,^32^ Denmark,^40^ Japan,^37^ The Netherlands^34^ and Thailand.^45^ All studies were of adults. Fourteen studies reported on funding; types of funding sources included academic research bodies, health-related institutions, commercial organisations and pharmaceutical companies.

**Table 1:** Summary characteristics and outcomes of alcohol reduction interventions (further details are presented in online supplementary files 2–8)

				AUDIT total scores			FAST total scores						
Study ID	Study characteristics	Description	Baseline behaviour	Mean change from baseline*	95% CI	N	Mean change from baseline*	SD	N	Global quality rating†	Effectiveness‡	Cost-effectiveness	Differential effects§
Dhital *et al*^27^	Design: RCT Duration: 12 weeks¶ Country: UK Number of pharmacies: 16 Number of participants: 407 Mean age: I:39.6; C: 40.5 % female: I: 47.8; C: 43.6	Brief alcohol advice	AUDIT Scores: 11.93 (SD 3.24)	−0.11	−0.82 to 0.61	168				Strong	↔	NR	NR
		Usual care control	AUDIT Scores: 11.53 (SD 3.19)	−0.74 p=0.24	−1.47 to 0.00	158							
Watson and Stewart^28^	Design: RCT Duration: 26 weeks Country: UK Number of Pharmacies : 20 Number of participants: 69 Mean age: NR % female: I: 48.1; C: 57.1	Brief alcohol advice	FAST score ≥3: 29.2%				2.25 0.50	3.20 0.71	4M 2F	Weak	↔	Cost analysis only	NR
		Usual care control	FAST score ≥3: 24.6%				−1.25 0.75 NS	2.87 1.67	4M 8F				

*p Values were extracted directly from the study papers and relate to between group differences.

†Global rating: ‘strong’=no ‘weak’ ratings, ‘moderate’=one ‘weak’ rating and ‘weak’=two or more ‘weak’ ratings.

‡Effectiveness was assessed using between group differences.

§Differential effects: age, gender, ethnicity or socioeconomic status (education, income, occupation, social class, deprivation or poverty).

¶From baseline to last follow-up.

↑, intervention effective; ↓, intervention not effective; ↔, no statistically significant between group difference; AUDIT, Alcohol Use Disorders Identification Test; C, control group; F, female; FAST, Fast Alcohol Screening Tool; I, intervention group; M, male; NR, not reported; NS, non-significant; RCT, randomised controlled trial.

**Table 2:** Summary characteristics and outcomes of smoking cessation interventions (further details are presented in online supplementary files 2–8)

Study ID	Study characteristics	Description	Baseline behaviour	Quit rate*	Global quality rating†	Effectiveness‡	Cost-effectiveness***	Differential effects§
Bauld *et al*^29^	Design: CBA Duration: 52 weeks¶ Country: UK Number of pharmacies: >200 Number of participants: 1785 Mean age: I: 44.0; C: 49.8 % female: I: 56.5; C: 65.5	Individual pharmacy-based NHS smoking cessation service + NRT	21+ cigarettes/day: 396 (40.1%)	38/1374 (2.8%)	Weak	?	Yes both services compared to control	NR
		Group community-based NHS smoking cessation service + NRT	21+ cigarettes/day: 169 (41.6%)	26/411 (6.3%) p=0.001				
Bock *et al*^30^	Design: RCT Duration: 26 weeks Country: USA Number of pharmacies: 2 Number of participants: 299 Mean age: I1: 45.5; I2: 46.5; C: 42.3 % female: 59.0	Smoking cessation training for pharmacists + tailored counselling using computer software + NRT	Number of cigarettes smoked/day: 18.2; Fagerström score: 5.3	28/100 (28.0%)	Moderate	↑	NR	NR
		Smoking cessation training for pharmacists + tailored counselling using computer software	Number of cigarettes smoked/day: 17.7; Fagerström score: 5.1	15/100 (15.0%)				
		Observation only control (not randomised)	Number of cigarettes smoked/day: 13.8; Fagerström score: 4.9	8/99 (8.1%) p<0.01				
Burford *et al*^31^	Design: RCT Duration:26 weeks Country:Australia Number of pharmacies:8 Number of participants:160 Mean age:I:24.2; C:25.1 % female: I:68.7; C:56.2	Smoking cessation advice + computer-generated photoageing	Fagerström score: 2.87; >21 cigarettes/day smoked: 10%	11/80 (13.8%)	Moderate	↑	Yes	NR
		Smoking cessation advice	Fagerström score: 2.96; >21 cigarettes/day smoked: 15%	1/80 (1.3%) p=0.003				
Costello *et al*^32^	Design: RCT Duration:5 weeks Country:Canada Number of pharmacies:98 Number of participants:6987 Mean age:NR % female: I:54.4; C:54.9	1 week then fortnightly visit for NRT plus 3 sessions brief behavioural counselling	HSI ≥3: 91.8%	612/3503 (17.5%)	Weak	↔	NR	NR
		5 weeks NRT at initial visit plus 1 session brief behavioural counselling	HSI ≥ 3: 91.4%	604/3350 (18.0%) p=0.4				
Crealey *et al* 1990<CE: Please check year is not matching with reference list.>	Design:nRCT Duration:26 weeks Country:UK Number of pharmacies:2 Number of participants:169 Mean age:NR % female: NR	Behavioural support, 67% (35/52) nicotine gum	NR	24/52 (46.2%)	Weak	↑	Yes	NR
		Nicotine gum only	NR	3/48 (6.3%)				
		Control (expressed wish to stop smoking)	NR	0/60 (0%) p<0.01 (I vs C)				
Hoving *et al*^34^	Design: RCT Duration:52 weeks Country:Netherlands Number of pharmacies:65 Number of participants:545 Mean age:I:46; C:47 % female: I:53; C:54	Computer-generated tailored advice	Number of cigarettes smoked/day: 22	2/256 (0.8%)	Strong	↔	NR	NR
		‘Thank you’ letter control	Number of cigarettes smoked/day: 21	2/289 (0.7%) NS				
Howard-Pitney *et al*^35^	Design: RCT Duration:26 weeks Country:USA Number of pharmacies:5 Number of participants:410 Mean age:I:36.3; C:34.7 % female: I:1; C:1	Advice and support + nicotine patch	Number of cans chewed/week: 3.9	78/206 (37.9%)	Moderate	↔	NR	NR
		Advice and support + placebo patch	Number of cans chewed/week: 4.1	69/204 (33.8%) p<0.40				
Maguire *et al*^36^	Design: RCT Duration:52 weeks Country:UK Number of pharmacies:51 Number of participants:484 Mean age:I:42; C:38 % female: I:40; C:44	Behavioural support, 87% (230/265) NRT	Number of participants 10–20 cigarettes/day: 197/265	38/265 (14.3%)	Weak	↑	NR	NR
		Ad hoc advice, 84% (183/219) NRT	Number of participants 10–20 cigarettes/day: 121/219	6/219 (2.7%) p < 0.001				
Mochizuki *et al*^37^	Design: RCT Duration:12 weeks Country:Japan Number of pharmacies:14 Number of participants:28 Mean age:I:44.1; C:49.1 % female: I:18.2; C:18.8	Nicotine gum plus advice on usage, initial and follow-up cessation advice	Number of cigarettes smoked/day: 23.0; Fagerström score: 4.56	5/11 (45.5%)	Strong	↔	NR	NR
		Nicotine gum plus advise on usage	Number of cigarettes smoked/day: 25.7; Fagerström score: 6.31	5/16 (31.3%) OR=1.83, NS				
Sinclair *et al*^38^	Design: RCT Duration:36 weeks Country:UK Number of pharmacies:62 Number of participants:492 Mean age:I:41.7; C:41.5 % female: I:61.2; C:62.7	Training pharmacists/assistants in smoking cessation behaviour change + NRT	Fagerström score: 5.2	26/217 (12.0%)	Strong	↔	Yes	NR
		Standard professional pharmacy support + NRT	Fagerström score: 5.2	19/257 (7.4%) p=0.089				
Sonderskov *et al*^40^	Design: RCT Duration:26 weeks Country:Denmark Number of pharmacies:42 Number of participants:522 Mean age:I(21 mg):39.1; C(21 mg):39.9; I(14 mg):38.2; C(14 mg):38.9 % female: I(21 mg):47.5; C(21 mg):52.5; I(14 mg):51.7; C(14 mg):48.3	21 mg nicotine patches	Fagerström score: 7.0	15/132 (11.4%)	Strong	↑ 21 mg; ↔ 14 mg	NR	No (gender)
		Placebo	Fagerström score: 8.1	6/142 (4.2%) p<0.05				
		14 mg nicotine patches	Fagerström score: 6.1	27/119 (22.7%)				
		Placebo	Fagerström score: 6.1	23/125 (18.4%) NS				
Vial *et al*^39^	Design: RCT Duration:52 weeks Country:Australia Number of pharmacies:9 Number of participants:102 Mean age:51.0 % female: I1:41; I2:54; C:36	Pharmacy-based nicotine patches plus weekly counselling	Fagerström score: 5.79	4/21 (19.0%)	Weak	↔	NR	NR
		Hospital outpatient clinic nicotine patches plus weekly counselling	Fagerström score: 5.94	5/21 (23.8%)				
		Minimal intervention (written and verbal information at baseline)	Fagerström score: 6.33	1/22 (4.5%) NS				

*p Values were extracted directly from the study papers and relate to between group difference.

†Global rating: ‘strong’=no ‘weak’ ratings, ‘moderate’=one ‘weak’ rating and ‘weak’=two or more ‘weak’ ratings.

‡effectiveness was assessed using between group differences.

§Differential effects: age, gender, ethnicity or socioeconomic status (education, income, occupation, social class, deprivation or poverty).

¶From baseline to last follow-up.

?, Unable to assess effectiveness/cost-effectiveness; ↑, intervention effective; ↓, intervention not effective; ↔, no statistically significant between group difference; C, control group; CBA, controlled before-after study; Fagerström score, 0–10, higher score=greater nicotine dependence; HIS, Heaviness of Smoking Index, higher score indicates greater number of cigarettes smoked per day and smoking first cigarette within 5 min of waking; I, intervention group; NHS, National Health Service; NR, not reported; nRCT, non-randomised controlled trial; NRT, nicotine replacement therapy; NS, non-significant; RCT, randomised controlled trial.

**Table 3:** Summary characteristics and outcomes of weight management interventions (further details are presented in online supplementary files 2–8

				BMI (kg/m^2^)		WC (cm)		WT (kg)					
Study ID	Study characteristics	N	Description	Mean change from baseline§	SD/95% CI	Mean change from baseline§	SD	Mean change from baseline§	SD/95% CI	Global quality rating*	Effectiveness†	Cost-effectiveness	Differential effects‡
Ahrens *et al*^41^ 2011	Design: RCT Duration: 22 weeks¶ Country: USA Number of pharmacies: 1 Number of participants: 95 Mean age: I: 47.6; C: 47.8 % female: 87 Baseline BMI: I: 29.5; C: 29.0	45	Meal replacement diet	NR	NR	−8.08	NR	−5.6	NR	Weak	↔	NR	NR
		43	Low calorie diet	NR	NR	−7.82	NR	−5.2	NR				
Bush *et al*^42^	Design: CBA Duration: 15 weeks¶ Country: UK Number of pharmacies: 12 Number of participants: 451 Mean age: I: 38.9; C: 42.6 % female: I: 87; C: 85 Baseline BMI: I: 33.0; C: 35.6	60	Pharmacy-based diet + physical activity	−1.3	0.4	−6.5	1.6	−3.4	1.1	Weak	?	Unclear which service was more cost effective	Yes, demographics of participants differed significantly between settings
		22	GP-based diet + physical activity	−0.8	0.7	−4.9	2.6	−2.3	1.9				
Jolly *et al*^43^**	Design: RCT Duration: 52 weeks¶ Country: UK Number of pharmacies:NR Number of participants: 740 Mean age: Ph: 48.9; Ex: 49.7; WW: 50.7; SW: 48.8;RC: 48.8; NHS SD: 48.8; GP: 50.5; POC: 47.5 % female: Ph: 73; Ex: 75; WW: 72; SW: 65; RC: 69; NHS SD: 64; GP: 67; POC: 70 Baseline BMI: P: 33.4; Ex: 33.9; WW: 34.0; SW: 33.8; RC: 33.4; NHS SD: 33.8; GP: 33.1; POC: 33.4	70	Pharmacy-based diet + physical activity	−0.31	−0.7 to 0.0	NR	NR	−0.66	−1.7 to 0.4	Moderate	↔	Cost analysis only, commercial organisations lower cost than GP and pharmacy-based services	No (gender)
		100	Exercise only control	−0.45	−0.8 to −0.1	NR	NR	−1.08	−2.1 to −0.1		‡		
		100	Weight Watchers	−1.17	−1.7 to −0.7	NR	NR	−3.46	−4.8 to −2.1		↑		
		100	Slimming World	−0.71	−1.0 to −0.4	NR	NR	−1.89	−2.9 to −0.9		↔		
		100	Rosemary Conley	−0.75	−1.1 to −0.3	NR	NR	−2.12	−3.4 to −0.9		↔		
		100	NHS Size Down	−0.67	−1.0 to −0.3	NR	NR	−2.45	−3.6 to −1.3		↔		
		70	GP	−0.32	−0.7 to 0.1	NR	NR	−0.83	−2.0 to 0.4		↔		
		100	Participants own choice	−0.90	−1.3 to −0.5	NR	NR	−2.15	−3.4 to −0.9		↔		
Malone and Alger-Mayer^44^	Design: nRCT Duration: 26 weeks¶ Country: USA Number of pharmacies: NR Number of participants: 30 Mean age: I: 44.9; C: 42.8 % female: I: 93; C: 80 Baseline BMI: I: 48.3; C: 42.8	15	Pharmacist support + orlistat + usual outpatient care	NR	NR	NR	NR	−3.5	2.9	Weak	↔	NR	NR
		15	orlistat + usual outpatient care	NR	NR	NR	NR	−3.0	5.2				
Phimarn *et al*^45^	Design: RCT Duration: 16 weeks¶ Country: Thailand Number of pharmac ies: 1 Number of participants: 66 Mean age: I: 60.1; C: 59.1 % female: I: 75.8; C: 84.8 Baseline BMI: I: 27.5; C: 27.7	33	Pharmacist individual support	−0.8	0.07	0.1	0.03	−0.82	0.29	Strong	↔	NR	NR
		33	Primary care unit group support	0.19	0.04	−0.28	0.08	0.92	0.19				

*Global rating: ‘strong’=no ‘weak’ ratings, ‘moderate’=one ‘weak’ rating and ‘weak’=two or more ‘weak’ ratings.

†Effectiveness was assessed using between group differences.

‡Differential effects: age, gender, ethnicity or socioeconomic status (education, income, occupation, social class, deprivation or poverty).

**All intervention groups in the Jolly trial were compared to the exercise only control group (intervention groups were not directly compared).

§p Values were extracted directly from the study papers and relate to between group differences.

¶From baseline to last follow-up.

↓, intervention not effective; ↑, intervention effective; ↔, no statistically significant between group difference; ?, unable to assess effectiveness/cost-effectiveness; BMI, body mass index; C, control group; CBA, controlled before-after study; Ex, exercise only control; GP, general practitioner; NHS SD, NHS Size Down; NHS, National Health Service; NR, not reported; nRCT, non-randomised controlled trial; NS, non-significant; Ph, Pharmacy-based diet + physical activity; POC, participants own choice; RC, Rosemary Conley; RCT, randomised controlled trial; SW, Slimming World; WC, waist circumference; WT, weight; WW, Weight Watchers.

Three studies^29^ ^42^ ^43^ recruited participants from areas of high deprivation, and compared a pharmacy-based setting with other settings. Twelve studies recruited participants within the community pharmacy; other recruitment settings included hospital/primary care units, via telephone and a community health centre. Types of pharmacies included single outlets, small chains and large chains; set in rural, urban and a combination of both geographical settings. The number of pharmacies included within each study ranged from one to over 200. Participant sample size ranged from 28 to around 7000, comprising approximately 13 500 service users in total. Mean age ranged from 24 to 60 years; there was a majority of females across all studies, particularly in the weight management studies. Duration of follow-up ranged from 5 to 56 weeks.

In terms of data analysis, only four studies assessed whether sociodemographic variables moderated the effect of interventions; four studies^28^ ^38^ ^42^ ^43^ assessed any differential effects of gender, and one of these also assessed age.^42^ No study assessed any differential effects of SES. Few studies used regression analysis to assess the influence of sociodemographic or socioeconomic variables on change from baseline, as potential predictors of outcomes within intervention groups, or to explain retention.

### Quality assessment

The studies were assessed for quality using six criteria and assigned a global rating; six studies were rated ‘strong’, four studies ‘moderate’ and nine studies ‘weak’ (see online supplementary file 3). Participants were not obtained from a randomly selected sample in any of the studies. Five studies reported a low attrition rate, with follow-up of at least 80% of participants. Only five studies were sufficiently powered. Six studies conducted intention-to-treat analyses. Fifteen studies imputed data from baseline or last follow-up, or made assumptions about dropouts (eg, assumed that dropouts had not stopped smoking/not lost weight). Six studies used hierarchical modelling techniques to adjust for potential pharmacy or pharmacist-level effects on individual participant outcomes. None of the studies reported details about whether the intervention was delivered as intended, for example, by observation of sessions, quality control audits, or staff and researcher records.

### Implementation of the interventions

Very few studies reported any degree of consultation or collaboration, with stakeholders as part of the planning process, or during delivery of the intervention (see online supplementary file 4). Both the brief alcohol reduction interventions consulted with pharmacists during the planning stages.^28^ ^27^ The smoking cessation study by Hoving *et al*^34^ collaborated with a national charity on smoking and health, and together they developed the intervention. The smoking cessation study by Costello *et al*^32^ was nested within a ‘host’ study called ‘STOP’, which collaborated with different community and regional partners in many different ways during the planning and delivery of the intervention. In the majority of interventions, regardless of their target behavioural or health outcome, pharmacists received reimbursement for providing the intervention; this appears important in order for the intervention to be sustainable.^32^ ^36^

### Organisation and delivery of the interventions

Sixteen interventions were delivered by the community pharmacy staff; one photoageing intervention^31^ was delivered by a research pharmacist employed by the local university in collaboration with the community pharmacist, who delivered standard smoking cessation advice (see online supplementary file 5). Another smoking cessation intervention was conducted by a research pharmacist as part of an MSc project; the research pharmacist delivered the hospital-based intervention programme, and the community pharmacists delivered the community-based intervention programme.^39^ One smoking cessation intervention involved the postal delivery of a computer-generated letter.^34^ Most studies included standardised staff training, although this was usually brief (ranging from 2 h to 2 days). Two smoking cessation studies mentioned they also included role play as part of the training,^30^ ^35^ and two weight management studies reported ‘practical tasks’ as part of the training.^43^ ^45^

In terms of quality assurance, one alcohol reduction intervention provided a 2 h evening follow-up training session during the intervention to address challenges and share learning across the pharmacists who were delivering the intervention.^27^ In two smoking cessation studies,^33^ ^36^ a researcher visited the pharmacists after the group training session, to provide support and to address any queries they had in implementing the training. In one smoking cessation study that was organised by a pharmaceutical company,^40^ the company contacted pharmacies at least once a week during the intervention.

### Behaviour change strategies used in the interventions

Seven studies reported that a behavioural theory/model informed the intervention and provided details of behaviour change strategies used; six studies only reported details of behaviour change strategies used; six studies reported no relevant information (see online supplementary file 6). The most commonly reported theoretical model was the Transtheoretical (‘Stages of Change’) Model, which was reported by six studies; motivational interviewing was reported by five studies. The descriptions available did not allow for the coding of specific aspects of theory and behavioural content. Using the Behaviour Change Wheel,^18^ the intervention functions of the majority of interventions were ‘education’ and ‘enablement’. In addition, interventions that included the provision of NRT or commercial weight management programmes or products free of charge, were also deemed to include ‘incentivisation’. Using the policy category of the Behaviour Change Wheel,^18^ all the interventions were categorised as ‘service provision’. Six of these interventions also included ‘communication/marketing’. No other policy categories were identified. Using the Nuffield intervention ladder,^19^ most interventions were coded as ‘enable choice’.

## Effects of interventions

### Alcohol reduction interventions (n=2)

There were two RCTs of brief alcohol reduction interventions (table 1) compared with usual care or leaflet-only control (see online supplementary files 7 and 8). One RCT^27^ used the Alcohol Use Disorders Identification Test total scores (AUDIT), and reported a baseline AUDIT score of 11.93. The other RCT^28^ used the Fast Alcohol Screening Tool (FAST), and reported 29.2% of participants scoring ≥ 3 at baseline. Possible ‘harmful or hazardous’ alcohol consumption, but not alcohol dependence is indicated with an AUDIT score 8–19 or a FAST score of 3–16. Global quality ratings were ‘strong’ for one study^27^ and ‘weak’ for the other,^28^ which was a small pilot study. Both studies involved one-to-one contact with the pharmacist. Dhital *et al*^27^ encouraged self-directed behaviour change; the intervention included reflection and feedback of the AUDIT score.

#### Behavioural outcomes

Neither intervention significantly reduced alcohol scores compared with control. At 12 weeks, the AUDIT total change score did not differ significantly between the two groups and did not change significantly between baseline and follow-up in either group. Twelve-week AUDIT between group difference, adjusted for pharmacist gender, age, ethnicity and education, was −0.57 (95% CI −1.59 to 0.45). There was no significant difference between FAST score for the intervention group compared with control at 3 or 6 months, adjusted for baseline FAST: the difference between groups was −1.84 (95% CI −4.49 to 0.82). At 6 months, there was substantially lower follow-up of intervention participants (22.2%) compared with control participants (33.3%).

#### Costs

Cost-effectiveness of community pharmacy-based brief alcohol reduction interventions cannot be ascertained; only one pilot study^28^ reported direct intervention costs.

#### Differential effects by demographic or socioeconomic factors

One pilot study^28^ reported change in FAST scores by gender within the intervention and control groups. However, the study was not powered to detect differences between the two groups.

### Smoking cessation interventions (n=12)

There were 10 RCTs,^30–32^ ^34–40^ 1 nRCT^33^ and 1 CBA^29^ of smoking cessation interventions (table 2). Global quality ratings were ‘strong’ for four studies, ‘moderate’ for three studies, and ‘weak’ for five studies. Eleven studies carried out analyses with the assumption that those lost to follow-up had not stopped smoking. Half (6/12) the smoking cessation interventions relied on self-reported change in smoking behaviours,^32^ ^34^ ^37^ ^38–40^ and half used biochemical measures (carbon monoxide (CO) or cotinine levels).^29–31^ ^33^ ^35^ ^36^

Ten studies included NRT (in either the intervention or control group or both).^29^ ^30^ ^32^ ^33^ ^35–40^ Seven studies evaluated some form of behavioural support.^30^ ^32–34^ ^36–38^ Two studies evaluated the effect of intervention setting; one study assessed behavioural support plus NRT provided in a hospital outpatient setting compared with pharmacy setting.^39^ Another compared individual pharmacy-based behavioural support plus NRT with group-support provided in a community setting.^29^ One study^31^ evaluated the effect of a photoageing intervention.

Despite a variety of different components being evaluated within the individual interventions, the studies were grouped together to assess the effectiveness of any type of community pharmacy-delivered intervention for smoking cessation compared with either an active control or a non-active/usual care comparator. ‘Usual care’ varied between studies but was, in general, a minimal intervention, such as observation only, ad hoc smoking cessation advice or a thank you letter. However, in one study, the control group received placebo nicotine patches^40^ and in two studies the control group received standard cessation advice plus NRT.^36^ ^38^

#### Behavioural outcomes

Five of the 12 studies demonstrated effectiveness compared with control. In addition, Bauld *et al*^29^ evaluated one-to-one pharmacist support with group-based smoking cessation clinics based in the community; the group-based service attracted fewer clients but was more effective.

The five effective studies included:

- An American RCT^30^ of additional training to pharmacists to enable them to provide a tailored counselling service with and without NRT, compared with a non-randomised control group that received observation only, showed a significant increase in validated 7-day point prevalence at 6 months (28% for counselling and NRT, 15% for counselling, 8% for control).
- An Australian RCT^31^ of a computer-generated photoageing service (demonstrating the detrimental effects on facial physical appearance of smoking) in addition to standard smoking cessation advice from a pharmacist, was effective in stopping young people (mean age 24 years) smoking compared to control using CO-validated measures (13.8% n=22/80 vs 1.3% n=11/80) at 6 months.
- A cost-effectiveness study^33^ in two UK pharmacies compared a behavioural intervention group based on the Pharmacist Action on Smoking (PAS) model with a control group that received nicotine gum, and another control group who expressed a wish to stop smoking. At 6 months, there was a statistically significant difference in cessation rates between intervention and control groups. Six-month CO-verified abstinence was 46% in the intervention group, 6% in the nicotine gum control group, and 0% in the control group that expressed a wish to stop smoking.
- A UK RCT^36^ compared an intervention based on the PAS model to ad hoc smoking cessation advice; over 80% in each group also had NRT. The PAS intervention significantly increased validated smoking cessation compared with control at 12 months (14.3% vs 2.7%).
- A Danish study^40^ evaluated the effect of two different strengths of nicotine patches compared to placebo. Those smoking ≥20/day at baseline were randomised to 21 mg patches or placebo, those smoking <20/day at baseline were randomised to 14 mg patches or placebo. Self-reported point prevalence included participants who had one episode of smoking (<6 days). At 26 weeks, the intervention was effective for those smoking ≥20/day at baseline (11% vs 4.2%) but not effective for lighter smokers (22.7% vs 18.4%) compared with the respective placebo groups.

#### Meta-regression and meta-analysis

**Figure 2:**
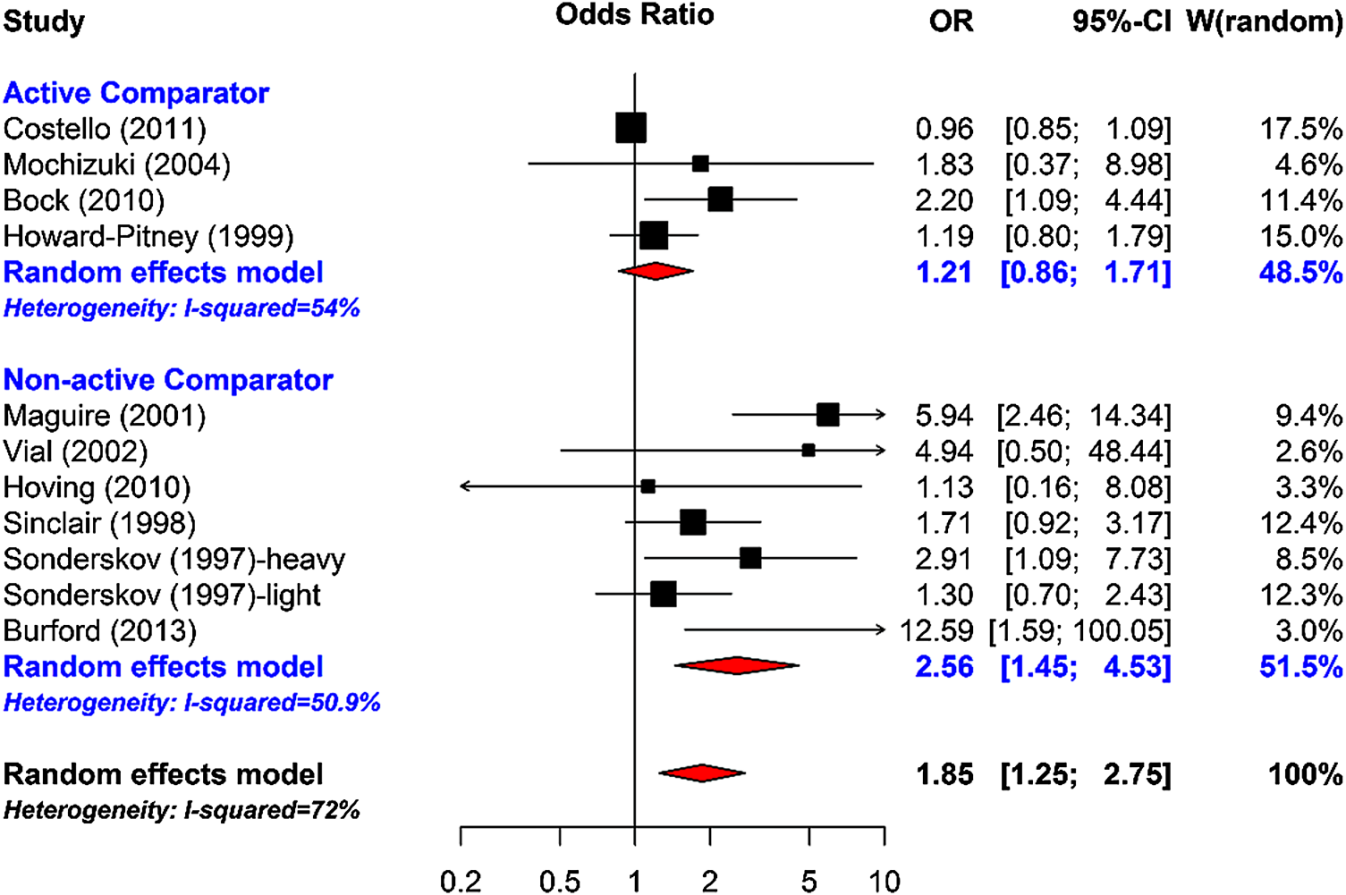
Meta-analysis of smoking cessation accounting for whether active comparator or non-active comparator.

Meta-regression of ORs of smoking cessation between the intervention and the control groups was undertaken; in model 1, a random effects model was fitted including all the RCTs. The pooled OR for the intervention effects was 1.85 (95% CI 1.25 to 2.75), an indication of the positive effect of the interventions on smoking cessation. However, there was 72% unexplained differences between the studies. In model 2, a meta-regression model was fitted accounting for whether a study had an active comparator or non-active/usual care comparator. The pooled ORs were 1.21 (95% CI 0.86 to 1.71) and 2.56 (95% CI 1.45 to 4.53) for the active comparator or non-active/usual care comparator, respectively (figure 2).

**Figure 3:**
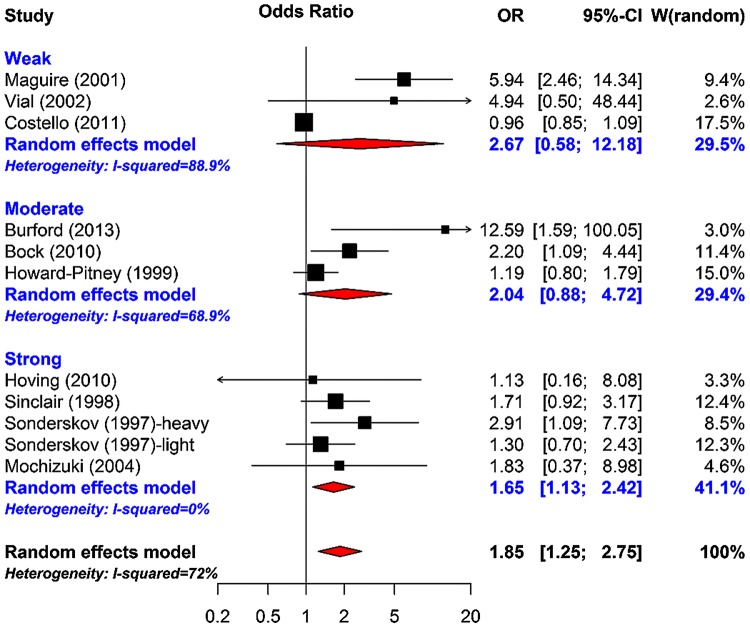
Meta-analysis of smoking cessation accounting for global quality rating.

As expected, there was a larger effect when compared with non-active/usual care comparator than with active comparator. The proportion of unexplained heterogeneity reduced to 52%. In model 3, a meta-regression model was fitted accounting for whether a study had an active comparator or a non-active/usual care comparator, and also the intervention duration; the unexplained heterogeneity reduced to 27.2% with a non-significant Q-statistic test (10.99, p <0.2026). In model 4, quality rating was accounted for; quality rating did not appear to contribute much to the model after accounting for intervention duration, and whether a study had an active comparator or a non-active/usual care comparator. Figure 3 shows a meta-analysis of smoking cessation accounting for global quality rating, and shows that most variations between studies are from studies rated as ‘moderate’ or ‘weak’ quality. A funnel plot demonstrated asymmetry, with larger studies showing effects closer to the null than smaller studies. Such a pattern is compatible with publication bias, on the assumption that smaller studies with uninteresting effects are withheld from publication. However, the funnel plot must be interpreted with caution, taking into account that it contains only 10 studies, which is the recommended study size threshold for creating such plots.^11^

#### Costs

Four studies reported cost-effectiveness analyses; the costs and benefits differed between the studies, and costs years ranged from 1995 to 2011, making comparisons across the analyses difficult. All four studies used quit rates observed within the trials, these ranged from 2.8% to 12% for UK pharmacist-based behavioural support with NRT.

By comparison with a self-quit attempt, the incremental cost per Quality Adjusted Life Year was £2600 for pharmacy one-to-one counselling, and £4800 for group community-based NHS smoking cessation service.^29^ Incremental Cost Effectiveness Ratios (ICER) per additional quitter ranged from £79 to £509 for pharmacist-based behavioural support with NRT. The ICER per additional quitter using photoageing was $A46 (Australian dollars 2011).^31^ In summary, three UK pharmacy-delivered interventions appeared cost effective across a range of quit rates, and an Australian photoageing intervention was cost effective compared to standard advice among young adults.

#### Differential effects by demographic or socioeconomic factors

A Danish study^40^ evaluated the effect of two different strengths of nicotine patches compared with placebo. There were no differences in smoking cessation rates between men and women according to starting dose and treatment.

### Weight management interventions (n=5)

There were three RCTs,^41^ ^43^ ^45^ one nRCT^44^ and one CBA^42^ of weight management interventions (table 3). Global ratings were ‘strong’ for one study, ‘moderate’ for one study, and ‘weak’ for three studies. Three studies^42^ ^43^ ^45^ compared a pharmacy-based intervention with similar interventions in other primary care settings, and commercial programmes in community settings. One study^41^ compared a meal replacement diet with a conventional low-energy diet (identical recommended total daily energy intake); both interventions were set in a pharmacy. One small study^44^ assessed the added value of community pharmacy support for an obesity management intervention that included orlistat and an outpatient nutrition programme.

#### Anthropometric outcomes

Three studies reported body mass index (BMI), three studies reported waist circumference (WC) and all five studies reported weight (WT). None of the studies found a significant difference in favour of a pharmacy-delivered intervention compared with the comparator, for any anthropometric outcome. However, all comparators are ‘active’ interventions (smoking cessation studies demonstrated larger effect when compared with non-active controls compared to active controls). One UK RCT^43^ compared seven groups (Weight Watchers, Slimming World, Rosemary Conley, Size Down an NHS community-based group, GP, Pharmacy, participants’ own choice to an exercise-only control group). This study compared each intervention group with a control group, and was not designed to directly compare the active interventions which were delivered across different settings. All, except the GP and pharmacy groups, resulted in significant weight loss at 1 year compared with baseline. Mean weight loss at 1 year, with baseline value used for imputation, was 0.8 kg (SD 4.7 kg) for primary care (GP and pharmacy) and 2.5 kg (SD 6.2 kg) for commercial programmes. Only the Weight Watchers group demonstrated significant weight loss at 1 year compared to control.

One CBA^42^ study compared diet and physical activity in a pharmacy to a GP-based intervention: both groups appeared to reduce BMI, WC and WT at follow-up (statistical significance not reported). Despite participants choosing the service, there was very high attrition (93%). One study^41^ demonstrated significant and similar amounts of weight loss from baseline to follow-up for participants in a meal replacement group, or a low-calorie diet group (both pharmacy-delivered). In another study, pharmacy-based support in addition to orlistat did not improve weight loss.^44^ Another study demonstrated no significant improvement in weight from baseline to follow-up for participants receiving group-based support in a primary care unit compared with individual support from a pharmacist.^45^

#### Costs

Two trials reported intervention costs, one of which also reported costs per kg weight lost.^42^ The Jolly *et al*^43^ trial reported similar costs (£112) for both the pharmacy group and the GP group; both settings had higher costs compared with commercial weight management programmes (£71–£77), the NHS community-based group costs fell in-between at £92.

A study^42^ of weight management programmes based in pharmacy or GP settings reported costs ((£126.90 per participant (n=183) in the pharmacy intervention and £100.60 per participant (n=268) in the GP intervention)), that were broadly similar to that of the pharmacy-based group in the Jolly trial. It is unclear which provider type delivered the intervention more cost-effectively; at session 12, the ICER (£ per kg per participant) cost −£8.29 through pharmacy providers (favours GP). Conversely, at the final session 15, the ICER was £2.91 through GP providers (favours Pharmacy).

#### Differential effects by demographic or socioeconomic factors

In a study of weight management programmes in various commercial, primary care and NHS settings, there was no statistically significant interaction between gender and the type of weight management programme.^43^ Bush *et al*^42^ compared a weight management programme set in pharmacies with the same programme set in GP surgeries. Female participants in GP surgeries lost a significantly larger proportion of their initial weight than female participants in pharmacies; participants aged 40–49 years lost a greater proportion of their initial weight at GP providers than at pharmacy providers.

## Discussion

Community pharmacy-delivered smoking cessation interventions including behavioural support and/or NRT, are effective and cost effective, particularly when compared with usual care. The pooled ORs for smoking cessation were 1.21 (95% CI 0.86 to 1.71) and 2.56 (95% CI 1.45 to 4.53) for active control and usual care, respectively. The heterogeneity of types of interventions precluded the ability to evaluate effectiveness by specific types of interventions. There was little evidence comparing pharmacy-delivered smoking cessation with smoking cessation delivered in other settings. This was in contrast with the majority of the weight management evidence which compared active interventions in various settings. Although there was no significant difference in weight loss between active interventions in different settings; pharmacy-based interventions produced similar amounts of weight loss (3–5 kg) from baseline to short-term follow-up (6 months or less), compared with active interventions in other primary care settings. There was insufficient evidence to evaluate community pharmacy-delivered interventions for alcohol reduction.

Community pharmacy-delivered smoking cessation interventions are cost-effective (compared to self-quit or standard care) across a range of quit rates. Cost-effectiveness of pharmacy-delivered weight management interventions is unclear; they have similar provider costs to those delivered in other primary care settings, which are greater than those delivered by commercial organisations. This review aimed to extract information on intervention costs and potential cost savings; however, it is not a review of economic evaluations and, as such, the methods of the economic evaluations are not critically appraised; we simply report the results of the economic evaluations that were conducted alongside included interventions.

Evidence suggests that duration of intervention is a predictor of effectiveness, for both the smoking cessation and weight loss studies; in the case of weight management, longer term weight loss may differ by setting. Regression analysis showed that duration of intervention accounted for some heterogeneity across the smoking cessation studies. One longer term weight management study compared interventions in a range of primary care and commercial settings; all except the pharmacy and GP groups resulted in significant weight loss at 1 year compared with baseline. The data reported in the studies identified for this review highlight the potential importance of predictors of success.

In terms of the effects of the interventions on health inequalities, some studies examined demographic and/or socioeconomic factors at recruitment stage, as potential predictors of outcomes within group, and/or to explain differences in retention. However, none of the studies reported subgroup analysis of treatment effect by SES. Three studies adopted a targeted approach to addressing inequality, by recruiting participants from deprived areas, and compared a pharmacy setting with other settings. In two of these ‘targeted’ studies (one smoking, one weight) the participants self-selected the service; there were demographic and socioeconomic differences between participants who self-selected treatment by setting. The evidence shows that the community pharmacy is an appropriate and feasible setting to deliver a range of public health interventions, and this setting has the potential to reach those most in need.

The original analysis plan included an examination of any potential relationships between intervention effectiveness and behaviour change strategies and/or models used, also whether any patterns existed between effective interventions and implementation factors (eg, pharmacist training or resource intensity) or the organisation and delivery of service (eg, stakeholder involvement). Unfortunately, the lack of relevant data reported meant that this analysis could not be undertaken. It is worth noting that the majority of interventions were implemented within the political context of extending the public health role of pharmacists.

These findings build on previous work; looking to the future, there is a Cochrane review(46) in progress with a broader remit than this review; it evaluates the effectiveness of a wider variety of health promotion, or health behaviour interventions, set in community pharmacy. This work will further develop the evidence base; in particular, evidence regarding implementation, organisation and delivery of other types of public health interventions which may be transferable to alcohol reduction, smoking cessation and weight management interventions.

*Implications for policy and practice*: The evidence shows a range of types of smoking cessation interventions that are feasible and effective within community pharmacies, and supports the commissioning of smoking cessation services in a community pharmacy setting. Smoking cessation services, contracted as a core part of the national contract, or part of a national ‘advanced’ service, may well be a reasonable option. In addition, the evidence shows that weight management services are no less effective compared with those delivered in other primary care settings. Therefore, given the potential reach, effectiveness and associated costs of these interventions, commissioners may consider using community pharmacies to help deliver some of their smoking cessation and weight management services.

*Implications for future research*: Further research is required to assess the effectiveness of community pharmacy-delivered alcohol reduction interventions, and more research is needed on the cost-effectiveness of community pharmacy-delivered alcohol, smoking and weight management interventions compared with other providers. It appears that the duration of intervention impacts on effectiveness, and this is likely to impact on cost-effectiveness. There is a lack of evidence regarding the effect of community pharmacy-based interventions for alcohol reduction, smoking cessation and weight loss on health inequalities. Targeted intervention studies provided some evidence that adults accessing pharmacies are a distinct group that may not access other primary care or commercial organisations. This evidence is derived from participants who self-selected the intervention and setting. However, more research is required on the reach of public health interventions delivered from a community pharmacy setting. Future studies should be sufficiently powered to detect small changes in behavioural and health outcomes and measure the equity effects of these small changes at a population level. Future studies should assess and report sociodemographic and socioeconomic variables, behaviour change strategies and models, implementation factors, the organisation and delivery of interventions, and costs.

## Conclusions

The evidence demonstrates that the community pharmacy is an appropriate and feasible setting to deliver a range of public health interventions. Community pharmacy-delivered smoking cessation interventions are effective and cost effective, particularly when compared with usual care. Evidence from a heterogeneous group of weight management interventions suggest that community pharmacy-delivered weight management interventions are as effective as similar interventions in other primary care settings, at least in the short term, and have similar provider costs. There is insufficient evidence to assess the effectiveness of community pharmacy-based interventions for alcohol reduction. The impact of community pharmacy-delivered interventions on inequalities in priority public health conditions is unclear.
