# Supplementary material for: Community pharmacy-delivered interventions for public health priorities: a systematic review of interventions for alcohol reduction, smoking cessation and weight management, including meta-analysis for smoking cessation
Source: BMJ Open. 2016 Feb 29;6(2):e009828. doi: 10.1136/bmjopen-2015-009828 (PMC4780058; doi:10.1136/bmjopen-2015-009828)
Supplement: Supplementary data [file bmjopen-2015-009828supp.pdf]

Supplementary evidence

Supplementary file 1: (MEDLINE, Ovid) Search strategy

#	**search term**
1	exp Community Pharmacy Services/
2	Pharmacies/
3	exp Pharmacists/
4	exp Pharmacists' Aides/
5	Pharmacy/
6	chemist.tw.
7	(communit$ adj7 pharmac$).tw.
8	(office$ adj7 pharmacy$).tw.
9	((pharmacy or pharmacist? or pharmacies) adj3 (community or counsel$ or advice or care)).tw.
10	(pharmacist? adj3 (front line or 'one to one' or face to face)).tw.
11	(pharmacist? or pharmacy or pharmacies).tw.
12	((pharmacist? or pharmacy) adj3 (aide or aides or assistant? or staff)).tw.
13	(Pharmacist? adj2 (care or delivered)).tw.
14	(pharmacist? adj3 (counsel$ or (patient? adj2 education$) or led or intervention? or public health or diagnos$)).tw.
15	or/1-14
16	exp Obesity/
17	exp Body Weight/
18	exp Body Weight Changes/
19	exp Weight Gain/ or exp Weight Loss/
20	(obese or obesity).tw.
21	overweight.tw.
22	weight.tw.
23	diet$.tw.
24	nutrition$.tw.
25	(physical$ adj activ$).tw.
26	exercise$.tw.
27	lifestyle$.tw.
28	(bmi$ or (body adj mass ind$)).tw.
29	(waist adj6 circumference$).tw.
30	((weight adj2 (control or reduction) adj2 (advice or counsel$ or program$ or intervention?)) or (weight adj manag$)).tw.
31	((overweight or obese or obesity) adj4 (Advice or counsel$ or intervention? or program$)).tw.

32	or/16-31
33	exp Smoking/ or exp Smoking Cessation/
34	nicotine.tw.
35	cigarette$.tw.
36	(nicotine replacement therapy or NRT).tw.
37	smoking cessation.tw.
38	smok$.tw.
39	exp "Tobacco Use Cessation"/
40	exp Smoking Cessation/
41	(smoking cessation or (quit$ adj2 smok$)).tw.
42	((reduce or reducing) adj3 ('tobacco use' or cigarette? or smoking or addiction)).tw.
43	or/33-42
44	alcohol.mp.
45	exp Alcohols/
46	exp Alcohol Drinking/
47	exp Alcoholism/
48	exp Drinking Behavior/
49	(drink$).tw.
50	beer.tw.
51	wine.tw.
52	ethanol.tw.
53	drunk.tw.
54	(addict$ or (alcohol adj2 (abus$ or misus$))).tw.
55	alcohol$.tw.
56	drunk$.tw.
57	intoxicat$.tw.
58	or/44-57
59	32 or 43 or 58
60	(animals not humans).mp.
61	59 not 60
62	15 and 60
63	limit 62 to humans

Supplementary file 2: Table of study characteristics of interventions

Study ID and funding source	Methods	Participants	Intervention	Outcomes
**Alcohol reduciton interventions**				
Dhital (2015)<br><br>**Funding source:** Hugh Linstead Fellowship Award; Royal Pharmaceutical Society of Great Britain and the Harold and Marjorie Moss Charitable Trust PhD award; Wellcome Trust Research Career Development fellowship (WT086516MA); Service Support Payment by North West London CLRN (UKCRN number 11920)	**Design:** RCT<br>**Aim:** To evaluate the effectiveness of a brief alcohol intervention by community pharmacists to reduce hazardous or harmful drinking<br>**Power:** Yes<br>**ITT:** No	**Age (years):** I: 39.6 C: 40.5<br>**Sex F (%):** I: 47.8 C: 43.6<br>**Ethnicity:** 53.8% White British<br>**SES indicator:** Age, Education Ethnicity, Gender;<br>Age and gender of pharmacists<br>**Baseline Audit Scores:** I: 11.93 (3.24) C: 11.53 (3.19)<br>**Population:** Adult (≥18 years) pharmacy clients with AUDIT score 8-19<br>**Number:** 407<br>**Intervention Setting:** 16 community Pharmacies in the London Borough of Hammersmith and Fulham<br>**Recruitment Setting:** Community Pharmacy<br>**Country:** London, UK	**Who delivered:** Pharmacists (n=17)<br>**Intervention:** Structured: Intervention aimed at promoting behaviour change.<br>10 minutes brief motivational discussion with pharmacist to encourage contemplation/change of drinking habits. Materials provided included "Units & You" booklet and "Unit/Calorie Calculator Wheel" and an alcohol services leaflet.<br>**Control/Other:** Leaflet given, "Alcohol: The Basics" – not expected to change behaviour<br>**Duration (months):** 3	AUDIT scores; proportions remaining hazardous or harmful drinkers (scoring ≥8);<br>3 sub-scale scores of the AUDIT (for consumption, problems and dependence);<br>EQ-5D
Watson & Stewart (2011)<br><br>**Funding source:** Chief Scientist Office, Scotland	**Design:** RCT (pilot cluster)<br>**Aim:** To examine the provision of a brief alcohol intervention in community pharmacies, in terms of practical considerations, recruitment of pharmacists and client, uptake,	**Age (years):** Adult clients (≥18 years)<br>**Sex F (%):** I: 48.1%, C: 57.1% F<br>**Ethnicity (% White**): I: 96.6. C: 100%<br>**SES Indicator:** Education, Employment status, Ethnicity, Gender, IMD, Marital status<br>**Baseline FAST score ≥3 (%):** I: 29.2, Control: 24.6<br>**Population:** Adult (≥18 years) pharmacy clients with FAST scores ≥ 3<br>**Number:** 69<br>**Intervention Setting:** Community Pharmacies in Grampian (n=20)	**Who delivered:** Pharmacists (n=10) and pharmacy staff (n= ≤10)<br>**Intervention:** Clients were provided with a brief alcohol intervention delivered by pharmacists specifically trained in brief alcohol intervention techniques<br>**Control/Other:** Clients were provided with standard healthy living leaflets<br>**Duration (months):** 6	FAST scores;<br>Self-reported alcohol consumption;<br>Number of alcohol-free days during an average week;<br>Barriers/facilitators to delivering intervention (by pharmacists);

Study ID and funding source	Methods	Participants	Intervention	Outcomes
	potential effectiveness and acceptability to pharmacists and clients.<br>**Power:** No<br>**ITT:** No	**Recruitment Setting:** Community Pharmacies<br>**Country:** Scotland, UK		Pharmacy users opinions;<br>Staff and training costs
**Smoking cessation interventions**				
Bauld (2011)<br><br>**Funding sources:** Glasgow Centre for Population Health, NHS Greater Glasgow and Clyde, and NHS Health Scotland	**Design:** CBA<br>**Aim:** To assess one-year outcomes and a cost-utility analysis of two NHS smoking cessation services. SC (Smoking Concerns) and SF (Starting Fresh)<br>**Power:** Unclear<br>**ITT:** No	**Age (years):** SF: 44, SC: 49.8<br>**Sex F (%):** SF: 56.5%, SC: 65.5% F<br>**Ethnicity:** NR<br>**SES Indicator:** Age, Gender, Area, Housing status, Eligibility for free prescriptions, Employment status, Education, SES group score, Marital status<br>**Baseline no of participants smoking 21+ cigarettes daily:** SF: n=396 (40.1%), SC: n=169 (41.6%)<br>**Population:** Smokers ≥16 years accessing stop smoking services<br>**Number:** 1979 (SF: 1508, SC: 471)<br>**Intervention Setting:** Over 200 Community pharmacies (90% in Glasgow Health Board area)<br>**Recruitment Setting:** Community pharmacies<br>**Country:** Glasgow, UK	**Who delivered:** Pharmacists and Pharmacist assistants (n=NR)<br>**Intervention:** Pharmacy based smoking cessation intervention Starting Fresh (SF) involving 12 weeks of one-to-one counselling with a pharmacist combined with the direct supply of Nicotine Replacement Therapy (NRT) (in most cases the 16-hour Nicorette patch)<br>**Control/Other**: Group-based support smoking cessation service, Smoking Concerns (SC) involved 7 weeks of Group Community-based behavioural counselling<br>**Duration (months):** 12	CO-validated quit rates;<br>CO-validated quit rates by socioeconomic group score and also by Scottish deprivation quintile;<br>Cost-utility analysis;<br>Self-reported quits;<br>Use of cessation aids
Bock (2010)<br><br>**Funding sources:** Grants from the National Institutes of Health, National Cancer Institute (CA099881) and National Institute on Drug Abuse	**Design:** RCT + non-random control<br>**Aim:** To test the effectiveness of a computer tailored smoking cessation intervention Exper_Quit (EQ) assisted pharmacist counselling group vs	**Age (years):** EQ group 1: 46.5. EQ + group 2: 45.5 and /Control group: 42.3<br>**Sex F (%):** 59% F<br>**Ethnicity:** 91% White<br>**SES indicator:** Age, Education, Ethnicity, Gender, Income<br>**Baseline no of cigarettes smoked/day:** EQ: 17.7 EQ+: 18.2, Control: 13.8<br>**Baseline Fagerström:** I1: 5.3; I2: 5.1; C: 4.9<br>**Population:** Adult (>18 years) pharmacy client,	**Who delivered:** Trained Pharmacists (n=6)<br>**Intervention**: Two intervention groups: 1) EQ assisted pharmacist counselling and 2) EQ plus 8 weeks of nicotine transdermal patch (EQ+) EQ is a computer-driven software system, “Exper_Quit” (EQ), that provided individually tailored interventions to patients who smoke	7-day point prevalence abstinence (verified by saliva cotinine);<br>Quit attempts;<br>Predictors of cessation

Study ID and funding source	Methods	Participants	Intervention	Outcomes
(DA022167)	Exper_Quit+ EQ plus 8 weeks of nicotine transdermal patch vs control<br>**Power:** Unclear<br>**ITT:** No	daily cigarette smoker (≥5 cigarettes/ day for ≥3 months)<br>**Number:** 299<br>**Intervention Setting:** Pharmacies located within 2 large urban community health centres<br>**Recruitment Setting:** Pharmacies<br>**Country:** USA	and matching tailored reports for pharmacists to help guide cessation counselling<br>**Control:** observation only control<br>**Duration (months):** 6	
Burford (2013)<br><br>**Funding sources:** NR	**Design:** RCT<br>**Aim:** To test an intervention based on personalised vivid illustrations (using APRIL software) of smokers face among young smokers (18-30 years) and to explore the value of an unfunded intervention within pharmacies<br>**Power:** Yes<br>**ITT:** Yes	**Age (years):** I: 24.2 C: 25.1<br>**Sex F (%):**<br>I: 68.7, C: 56.2%<br>**Ethnicity:** NR<br>**SES indicator:** Age, Education, Gender<br>**Baseline Fagerström score:** I: 2.87, C: 2.96<br>**Population:** Smokers aged 18-30 years accessing 8 metropolitan community pharmacies.<br>**Number:** 160<br>**Intervention Setting:** Community Pharmacies in Western Australia<br>**Recruitment Setting:** Community pharmacies<br>**Country:** Perth, Australia	**Who delivered:** Pharmacists (n=NR)<br>**Intervention:** To assess whether the use of APRIL (face aging software) plus standard care (2-minute smoking cessation advice from pharmacist had an impact on self-reported quit rates of young smokers (aged 18-30) confirmed by CO testing<br>**Control:** Standard care (standardised 2-minute smoking cessation advice)<br>**Duration (months):** 6	CO-validated quit rates;<br>Cost-effectiveness analysis;<br>Nicotine dependence (Fagerström scale);<br>Progression along Transtheoretical stages of change model;<br>Quit attempts;<br>Self-reported quit
Costello (2011)<br><br>**Funding source:** Ontario Ministry of Health Promotion	**Design:** RCT+ non-random control<br>**Aim:** To evaluate the effectiveness of two models of pharmacist-led behavioural counselling for smoking cessation support provided by community pharmacists that included NRT<br>**Power:** Yes<br>**ITT:** No	**Age (years):** ≥18 years<br>**Sex F (%):** Group A: 54.4, Group B: 54.9<br>**Ethnicity:** NR<br>**SES indicator:** Age, Education, Employment, Gender<br>**Baseline Heaviness of smoking Index score ≥ 3 (%):** Group A: 91.8, Group B: 91.4<br>**Population:** Ontario residents, ≥18, self-report current daily smokers of 10+ cigarettes/day and willing to make a quit attempt within the next 30 days<br>**Number:** 15.898 (6,987 unique participants)<br>**Intervention Setting:** Community pharmacies (n=98)<br>**Recruitment Setting:** Online	**Who delivered:** Pharmacists (n=113)<br>**Intervention:** Group A received 3-behavioural counselling sessions with a pharmacist and 5 weeks of free NRT. Group B received one individual counselling session plus 5 weeks of free NRT. Group B received all 5 weeks of NRT at their one session and group A received theirs over 3 sessions.<br>**Duration (weeks):** 5-12 weeks, mean 6.4	Self-reported 7-day point prevalence;<br>Predictors of abstinence

Study ID and funding source	Methods	Participants	Intervention	Outcomes
		**Country:** Ontario, Canada		
Crealey (1998)<br><br>**Funding source:** NR	**Design:** CBA<br>**Aim:** To determine the costs and effects associated with a community pharmacy-based smoking cessation programme in Northern Ireland, using the perspective of the payer in the main analysis<br>**Power:** Unclear<br>**ITT:** Unclear	**Age (years):** NR<br>**Sex F (%):** NR<br>**Ethnicity:** NR<br>**SES indicator:** NR<br>**Baseline measures:** NR<br>**Population:** 52 people entered the smoking-cessation programme (group 1), 48 bought nicotine gum and gave their address so that additional information could be sent and they could be followed-up (group 2), and 60 people who expressed a wish to stop smoking were chosen on the basis that they matched, by age, gender, social status and disease status, those in group 1<br>**Number:** 160<br>**Intervention Setting:** 2 community pharmacies<br>**Recruitment setting:** NR<br>**Country:** Belfast, Northern Ireland, UK	**Who delivered:** Pharmacists (n=NR)<br>**Intervention:** Smoking cessation advice from community pharmacist, the Pharmacist Action on Smoking (PAS) Model. Developed by the PAS group in association with the National Pharmaceutical Association (NPA) in the UK, a written 'contract' between the patient and pharmacist (including a 'stop date'), and a series of brief counselling meetings over approximately 6 months.<br>**Control:** Participants received normal type of *ad hoc*, non-formalised advice that is currently given in community pharmacies<br>**Duration (months):** 6	CO-validated quit rates;<br>Cost-effectiveness analysis
Hoving (2010)<br><br>**Funding source:** NR	**Design:** RCT<br>**Aim:** to test the effectiveness of a computer tailored smoking cessation intervention distributed through GPs and pharmacies<br>**Power:** Unclear<br>**ITT:** Yes	**Age (years):** I: 46, C: 47<br>**Sex F (%):** I: 53, C: 54<br>**Ethnicity:** NR<br>**SES Indicator:** Age, Education, Gender<br>**Baseline no cigarettes smoked per day (mean):** I: 22, C: 21<br>**Baseline stage of change (contemplator/preparer %):** I: 41/59, C: 41/50<br>**Population:** Smokers accessing pharmacies and who had smoked within the last 7 days, >18 years, motivated to quit<br>**Number:** 545<br>**Intervention Setting:** community pharmacies<br>**Recruitment Setting:** community pharmacies (n=65)<br>**Country:** Netherlands	**Who delivered:** Pharmacists (n=NR)<br>**Intervention:** Participants received computer-generated tailored advice/messages to aid smoking cessation.<br>**Control:** thank you letter only<br>**Duration (months):** 12	Self-reported continued abstinence;<br>Self-reported 7-day point prevalence;<br>quit attempts;<br>(only pharmacy intervention included because GP intervention outcomes reported at different time)

Study ID and funding source	Methods	Participants	Intervention	Outcomes
Howard-Pitney (1999)<br><br>**Funding source:** National Institutes of health Public Health Service, Grant from National Cancer Institute. Drug supply agreement with Pharmacia & Upjohn AB.	**Design:** RCT<br>**Aim:** to examine the efficacy of a treatment program combining nicotine patch with a minimal contact for chewing tobacco users behavioural intervention<br>**Power:** Unclear<br>**ITT:** Yes	**Age (years):** I: 36.3 C: 34.7<br>**Sex F (%):** I: 1, C: 1%<br>**Ethnicity:** I: 96, C: 93 % white<br>**SES Indicator:** Age, Education, Ethnicity, Gender<br>**Baseline no of cans chewed/week:** I: 3.9, C: 4.1<br>**Population:** Adult, non-smoking, chewing tobacco users of at least one can or pouch of chewing tobacco each week and scored 6 or higher on a 10-point scale rating their motivation to quit.<br>**Number:** 410<br>**Intervention Setting:** Community Pharmacy (n=NR)<br>**Recruitment Setting:** Telephone<br>**Country:** USA	**Who delivered:** Pharmacists (n=NR)<br>**Intervention:** 15mg nicotine patch plus behavioural treatment including 2 pharmacy visits, 2 support calls, and self-help materials.<br>**Control:** Placebo nicotine patch plus behavioural treatment plus behavioural treatment including 2 pharmacy visits, 2 support calls, and self-help materials.<br>**Duration (months):** 6	7-day point prevalence (verified by cotinine);<br>Self-reported 7-day point prevalence;<br>Self-reported relapse (first day chewed tobacco for 7 days in a row);<br>Predictors of relapse;<br>Side Effects
Maguire (2001)<br><br>**Funding sources:** Medical Research Council and the Northern Ireland Department of Health and Social Services	**Design:** RCT<br>**Aim:** To evaluate whether a structured community pharmacy-based smoking cessation programme (the PAS model - Pharmacists Action on Smoking) would give rise to a higher smoking cessation rate compared with ad hoc advice from pharmacists<br>**Power:** Unclear<br>**ITT:** Yes	**Age (years):** I: 42 C: 38<br>**Sex F (%):** I:40, C:44<br>**Ethnicity:** NR<br>**SES Indicator:** Age, Gender<br>**Baseline no of smoking ≥ 20 cigarettes per day:** I: 42, C: 53<br>**Population:** Smokers attending community pharmacies<br>**Number:** 484<br>**Intervention Setting:** Community Pharmacies<br>**Recruitment Setting:** Community pharmacies<br>**Country:** Northern Ireland and London, UK	**Who delivered:** Pharmacists (n=124)<br>**Intervention:** The PAS intervention involved a structured counselling programme, an information leaflet and a follow-up weekly for the first 4 weeks then monthly as needed. (Smokers allocated to the PAS group received a leaflet and a one to one interview using the PAS flip-chart. Follow-up advice given at weekly intervals for 4 weeks, then monthly for 3 months.<br>**Control:** Participants accessed normal pharmaceutical service (including the provision of NRT) provided by the pharmacist. Smokers were not counselled using the PAS flip-chart, not given a PAS leaflet and not asked to attend for follow-up	Abstinence (verified by urinary cotinine);<br>Self-reported abstinence;<br>Pharmacist opinion on service

Study ID and funding source	Methods	Participants	Intervention	Outcomes
			interviews<br>**Duration (months):** 12	
Mochizuki (2004)<br>***Note:*** *Full paper in Japanese /English abstract: Not all information available for extraction.*<br><br>**Funding source:** Unsure	**Design:** RCT<br>Aim: to evaluate whether pharmacists advice on smoking cessation would result in a higher smoking cessation rate using Nicorette<br>**Power:** No<br>**ITT:** No	**Age (years):** I: 44.1 C: 49.1<br>**Sex F (%):** I:18.2, C: 18.8<br>**Ethnicity:** NR<br>**SES indicator:** Age, Gender<br>**Baseline no of cigarettes smoked per day (mean):** I: 23, C: 25.7<br>**Baseline Fagerström:** I: 4.56 (2.13) vs C 6.31 (1.85)<br>**Population:** Smokers visiting pharmacies from March 1 2002 through to August 31st 2002, ≥20, desire to quit smoking and smoke at least 11 cigarettes a day for last year.<br>**Number:** 28<br>**Intervention Setting:** Community Pharmacies (n=14)<br>**Recruitment Setting:** Community pharmacies<br>**Country:** Tokyo, Kanagawa & Nagano, Japan	**Who delivered:** Pharmacists (n=NR)<br>**Intervention:** Smokers received both regular instructions on Nicorette use and smoking cessation advice at first sale then follow-up advice prior to starting cessation and 1, 3, 8 weeks and 3 months thereafter.<br>**Control:** Smokers received regular pharmacist instruction only.<br>**Duration (months):** 3	Self-reported abstinence; Ergogram
Sinclair (1998)<br><br>**Funding:** Department of Health Scottish Office	**Design:** RCT<br>**Aim:** To evaluate a training workshop for community pharmacy personnel to improve their counselling in smoking cessation based on stage-of-change model<br>**Power:** No<br>**ITT:** No	**Age (years):** I: 41.7, C: 41.5<br>**Sex F (%):** I: 61.2, C: 62.7<br>**Ethnicity:** NR<br>**SES indicator:** Age, Gender, IMD<br>**Baseline Fagerström:** I: 5.2, C: 5.2<br>**Population:** Pharmacy customers who smoked<br>**Number:** 492<br>**Intervention Setting:** Non-city community pharmacies (n=62)<br>**Recruitment setting:** Community pharmacy<br>**Country:** Aberdeen, Scotland, UK	**Who delivered:** Pharmacists (n=40) and Pharmacist Assistants (n=54)<br>**Intervention:** Pharmacist Support Model (to incorporate Stages of Change Model to improve counselling).<br>**Control:** Control group customers were asked to register and continued to receive standard professional support<br>**Duration (months):** 9	Self-reported continuous abstinence; Customer and pharmacy personnel perceptions; Self-reported point prevalence; Cost-effectiveness analysis
Sonderskov (1997)<br><br>**Funding:** Partly funded by Ciba-Geigy (Nicotine	**Design:** RCT<br>**Aim:** to estimate short-term smoking cessation rates among selected	**Age (years):** 38.2/38.9 (14-mg/day patch group/placebo) 39.1/39.9 (21-mg/day patch group/placebo)<br>**Sex F (%):** 51.7/48.3 (14-mg/day patch group/placebo) 47.5/52.5 (21-mg/day patch	**Who delivered:** Pharmacists (n=NR) and pharmacy staff (n=NR)<br>**Intervention:** Nicotine patches: Customers who smoked 20 cigarettes or more per day were	Self-reported point prevalence (no smoking during a 4-week treatment period or one

Study ID and funding source	Methods	Participants	Intervention	Outcomes
patch supplier) no further information given	customers of nicotine patches at a number of pharmacies in Denmark and to evaluate smoking cessation on a long-rem basis<br>**Power:** Unclear<br>**ITT:** No	group/placebo)<br>**Ethnicity:** NR<br>**SES indicator:** Age, Education, Gender<br>**Baseline Fagerström Score:** 6.1/6.1 (14-mg/day patch group/placebo) 7.0/8.1 (21-mg/day patch group/placebo)<br>**Population:** Pharmacy customers (>18 years) who smoked ≥10 cigarettes/day<br>**Number:** 522<br>**Intervention Setting:** Community Pharmacies (n=42)<br>**Recruitment setting:** Community pharmacies in Aarhus and Copenhagen, Denmark<br>**Country:** Denmark	randomized to use one 21-mg/day patch per day during the first 4 weeks equivalent to one treatment period (active patches release 21 mg of nicotine in 24 hours), 14-mg/day patches (14 mg of nicotine/24 hours) during the second 4-week treatment period and 7-mg/day patches (7mg of nicotine/24 hours) during the final 4 weeks. Smokers of fewer than 20 cigarettes per day used 14-mg/day patches during the first two treatment periods (8 weeks), and 7-mg/day patches during the final treatment period.<br>**Control:** Placebo patches<br>**Duration (months):** 6	episode of a slip defined as <6 days of smoking within a 4-week period);<br>Self-reported point prevalence;
Vial (2002)<br><br>**Funding source:** Anti-Cancer Foundation of South Australia, The Queen Elizabeth Hospital Research Foundation and University of South Australia	**Design:** RCT<br>**Aim:** To compare quit rates, initiated in hospital (as inpatient, on discharge) using nicotine patches and support, either in a hospital outpatients, or community pharmacy<br>**Power:** No<br>**ITT:** No	**Age (years) mean/range:** 51.5/23-81)<br>**Sex F (%):** Community pharmacy: 41 Hospital: 54 and Minimal Int: 36<br>**Ethnicity:** NR<br>**SES indicator: (income/education/occupation/area)**<br>**Baseline Fagerström score**: Pharmacy: 5.79, Hospital: 5.94, Minimal Int: 6.33<br>**Population:** Inpatients aged over 18 years who smoked 10 or more cigarettes per day<br>**Number:** 102 (Hospital: n=35, Community pharmacy: n=34 and Minimal Int: n=33)<br>**Intervention Setting:** Community pharmacy<br>**Recruitment setting:** Queen Elizabeth Hospital<br>**Country:** Adelaide, Australia	**Who delivered:** Pharmacists<br>**Intervention:** Initial consultation with research pharmacist and begin nicotine patch treatment then followed with weekly counselling visits with either researcher in outpatient clinic or a community-based pharmacist<br>**Control:** Minimal intervention received written materials and advice on smoking cessation<br>**Duration (months):** 12	Self-reported continuous abstinence;<br>Self-reported 7-day and 30-day point prevalence
**Weight management interventions**				
Ahrens RA (2003)<br><br>**Funding:** Grant	**Design:** RCT<br>**Aim:**<br>To compare a meal	**Age (years):** I: 47.6 C: 47.8<br>**Sex F (%):** 87<br>**Ethnicity:** NR	**Who delivered:** Community pharmacists (n=2)<br>**Intervention:** Phase 1: Meal	>7% initial body weight;<br>Blood pressure;

Study ID and funding source	Methods	Participants	Intervention	Outcomes
from the Slim-Fast nutrition institute, West Palm Beach	replacement (MR) program with a conventional reduced-calorie diet (RCD) for weight management using the pharmacy as the setting and the pharmacist as the point of contact for dietary advice.<br>**Power:** Unclear<br>**ITT:** No	**SES indicator:** Age, Gender<br>**Baseline BMI:** I: 29.5 C: 29.0<br>**Population:** Patients with BMI between 25kg/m² - 32kg/m², aged 35-65 years<br>**Number:** 95<br>**Intervention Setting:** Community Pharmacies (n=1)<br>**Recruitment setting:** Unclear<br>**Country:** USA	Replacement Diet (Slim Fast) MR (Intervention group) patients drank one shake per day and ate two sensible meals of their choice.<br>Phase 2: Patients told to self-regulate their caloric intake with the goal of maintaining their weight loss.<br>**Control:** Phase 1: Self-selected diet based on diabetic exchange Phase 2: Conventional reduced calorie diet whereby patients were instructed to return to a healthy diet of their choice and to control caloric intake as desired<br>**Duration:** 22 weeks	Cholesterol;<br>Triglycerides;<br>Waist circumference;<br>Weight
Bush (2011)<br><br>**Funding:** NHS Birmingham	**Design:** CBA<br>**Aim:** to reduce adult obesity levels; improve access to overweight and obesity management services in primary care; improve diet and nutrition; promote healthy weight and increased levels of physical activity; support patients to make lifestyle changes delivery<br>**Power:** No<br>**ITT:** No	**Age (years):** I: 38.9, C: 42.6<br>**Sex F (%):** I: 87, C: 85<br>**Ethnicity:** 4/5 Black and Minority Ethnic Groups<br>**SES indicator:** Age, Gender, Ethnicity, IMD, Area<br>**Baseline BMI:** I: 33.0, C: 35.6<br>**Population:** Obese patients >18 years, BMI > 30 kg/m2 (>25 kg/m2 in Asian patients) or >28 kg/m2 (>23.5 kg/m2 in Asian patients) in patients with co-morbidities<br>**Number:** 451<br>**Intervention Setting:** Community pharmacies within the Heart of Birmingham Teaching Primary Care Trust (n=12)<br>**Recruitment setting:** I: Community pharmacies and C: GP surgeries<br>**Country:** Birmingham, UK	**Who delivered:** Pharmacists (n=NR)<br>**Intervention:** Pharmacy delivered My Choice Weight management programme including; weekly weight and waist circumference measurements, lifestyle, behaviour, diet and activity assessment, food and exercise diary, realistic weight loss targets (maximum weekly weight loss of 0.5-1 kg with the aim of a 5-10% reduction on initial weight), realistic targets for lifestyle, healthy eating and physical activity<br>**Control/other:** GP delivered My Choice Weight management programme (including same components as above)<br>**Duration (months):** 9	% WT by sex, age, indices of multiple deprivation (IMD) and ethnicity;<br>BMI;<br>Cost-effectiveness analysis;<br>Health-related quality of life (Short Form-12);<br>Waist circumference;<br>Weight
Jolly (2011)<br><br>**Funding:** NHS South Birmingham	**Design:** RCT (8-arm)<br>**Aim:** to determine the effectiveness of a range of NHS and	**Age (years):** I: 48.94, C: 49.67<br>**Sex F (%):** I: 73, C: 75<br>**Ethnicity:** I: 87, C: 84% White British/Irish<br>**SES indicator:** Age, Gender, Ethnicity, IMD,	**Who delivered:** Pharmacist (n=NR) and various others depending on assigned arm.<br>**Intervention:** Pharmacy arm: 12	>5% body weight loss;<br>BMI;<br>Costs;

Study ID and funding source	Methods	Participants	Intervention	Outcomes
	commercial weight loss programmes in an unselected primary care population<br>**Power:** Yes<br>**ITT:** Yes	Area<br>**Baseline BMI:** I: 33.44, C: 33.88<br>**Population:** Obese or overweight men and women, >18 years with a comorbid disorder identified from GP records<br>**Number:** 740<br>**Intervention Setting:** Community pharmacies<br>**Recruitment setting:** Call centre (telephone) nurse-led recruitment<br>**Country:** Birmingham, UK	one-to-one weight management sessions in the pharmacy, key messages on diet and physical activity, behavioural assessment, goal setting, plans for change, dealing with resistance, enhancing motivation, and weight maintenance. It included both practical tasks and informational components.<br>**Other arms:**<br>Weight Watchers (WW),<br>Slimming World (SW),<br>Rosemary Conley (RC),<br>Group based dietetics-led programme (SD),<br>General Practice one-to-one counselling (GP); all 12 weeks in duration<br>**Control:** vouchers for 12 free sessions at a local authority run leisure centre (a council run facility open to all members of the public and usually consisting of a swimming pool, fitness suite, and other sports halls or courts). Participants were not given an appointment to attend and were given no individual advice or support on diet or physical activity.<br>**Duration (months):** 12	Physical activity; Weight
Malone (2003)<br><br>**Funding:** NR	**Design:** nRCT<br>**Aim:** To evaluate the impact of pharmacist support + usual care for patients who were prescribed orlistat and attending an	**Age (years):** I: 44.9, C: 42.8<br>**Sex F (%):** I: 93, C: 80<br>**Ethnicity:** NR<br>**SES indicator:** Age, Gender<br>**Baseline BMI:** I: 48.3, C: 42.8<br>**Population:** Patients from a hospital outpatient clinic who were waiting to be initiated on to	**Who delivered:** Pharmacist (n=8)<br>**Intervention:** Intervention group were familiarised with their local pharmacist to make contact when collecting their prescription for orlistat. At first visit, they had a consultation with the pharmacists;	Weight

Study ID and funding source	Methods	Participants	Intervention	Outcomes
	outpatient nutrition program, compared with just usual care (outpatient appointments every 4-6 weeks) terms of patient compliance with orlistat<br>**Power:** No<br>**ITT:** Yes	orlistat therapy<br>**Number:** 30<br>**Intervention Setting:** Community pharmacies in patients area of residence (8 pre-selected pharmacists trained)<br>**Recruitment setting:** University teaching hospital-based outpatient nutrition clinic<br>**Country:** USA	(involving support on weight loss/pharmacotherapy) they were encouraged by the pharmacist to sign up to the Xenicare support line (Roche). These patients returned to see the pharmacists after 2 weeks for a follow up consultation. Unclear what the protocol was for number and frequency of consultations after this time. This intervention was in addition to 'usual care' = 4-6 week appointments at outpatient clinic.<br>**Control:** Usual Care provided by the outpatient clinic<br>**Duration (months):** 6	
Phimarn (2013)<br><br>**Funding:** National Health Security Office (NHSO) Thailand Fund supported grant through Primary Care Practice Research Unit, Mahasarakham University	**Design:** RCT<br>**Aim:** Examine clinical outcomes, eating behaviours, and knowledge about being overweight and obesity, comparing the community pharmacy intervention with routine group weight management<br>**Power:** Yes<br>**ITT:** No	**Age (years):** I:60.09, C: 59.12<br>**Sex F (%):** I: 84.8, C: 75.8<br>**Ethnicity:** NR<br>**SES indicator:** Age, Education, Gender, Income, Marital status, Occupation<br>**Baseline BMI:** I:27.49, C: 27.74<br>**Population:** overweight and obese patients from one Primary Care Unit (PCU) diagnosed as overweight or obese by a doctor<br>**Number:** 75<br>**Intervention Setting:** single community pharmacy where there was an established network with one Primary Care Unit<br>**Recruitment setting:** one selected Primary Care Unit, Mahasarakham<br>**Country:** Thailand	**Who delivered:** Pharmacists (n=NR)<br>**Intervention:** 16 week intervention, sessions (lasting about 1 hour, one-to-one sessions provided by a pharmacist along with the weight loss handbook for self-study. Sessions provided at 0, 4, 8 and 16 weeks.<br>**Control:** group counselling with a focus on weight loss which was routinely provided by the PCU staff. Typically, all overweight and obese patients. Group sessions lasted approximately 1 h, covered information about healthy diet, principles of energy intake, food groups, portion size, and exercise. The group counselling sessions were provided at weeks 0, 4, 8, and 16<br>**Duration (months):** 4	BMI;<br>Theory of Planned Behaviour scores;<br>Waist circumference;<br>Weight

AUDIT: Alcohol Use Disorders Identification Test; BMI: body mass index; CBA: controlled before and after study; CO: carbon monoxide; EQ-5D: European Quality of Life-5 Dimensions questionnaire; Fast Alcohol Screening Tool (FAST); IMD: Index of Multiple Deprivation; ITT: intention-to-treat; NR: not reported; nRCT: non-randomised controlled trial; NRT: nicotine replacement therapy; RCT: randomised controlled trial; SES: socioeconomic status

Supplementary File 3: Quality Assessment

Study ID	Selection bias	Study design	Confounders	Blinding	Data collection methods	Withdrawals and dropouts	Global rating*
**Alcohol reduction**							
Dhital 2015	moderate	strong	strong	moderate	moderate	moderate	strong
Watson & Stewart 2011	weak	strong	weak	moderate	moderate	weak	weak
**Smoking cessation**							
Bauld 2011	weak	moderate	strong	moderate	strong	weak	weak
Bock 2010	weak	strong	moderate	moderate	strong	strong	moderate
Burford 2013	moderate	strong	strong	weak	strong	moderate	moderate
Costello 2011	weak	strong	strong	weak	moderate	weak	weak
Crealey 1998	weak	strong	weak	moderate	moderate	weak	weak
Hoving 2010	moderate	strong	strong	moderate	strong	moderate	strong
Howard-Pitney 1999	moderate	strong	weak	strong	strong	strong	moderate
Maguire 2001	moderate	strong	weak	weak	strong	strong	weak
Mochizuki 2004	moderate	strong	strong	moderate	moderate	moderate	strong
Sinclair 1998	moderate	strong	strong	moderate	strong	moderate	strong
Sonderskov 1997	moderate	strong	strong	moderate	moderate	strong	strong
Vial 2002	weak	strong	strong	weak	moderate	moderate	weak
**Weight management**							
Ahrens 2011	moderate	strong	strong	weak	strong	weak	weak
Bush 2011	moderate	moderate	weak	moderate	strong	weak	weak
Jolly 2011	weak	strong	strong	moderate	strong	moderate	moderate
Malone 2003	moderate	strong	weak	moderate	strong	weak	weak
Phimarn 2013	moderate	strong	strong	moderate	strong	strong	strong

* Global rating: ‘strong’ = no ‘weak’ ratings, ‘moderate’ = one ‘weak’ rating and ‘weak’ = two or more ‘weak’ ratings

Supplementary file 4: Implementation

Study	Implementation context[1]	Consultation/ collaboration processes during planning[2]	Consultation/ collaboration processes during deliver[3]	Sustainability[4] *(Note: In most studies, where relevant, pharmacists received reimbursement for providing the intervention)*
**Alcohol reduction**				
**Dhital 2015**	Political	Pharmacists were consulted in the planning of the trial regarding an acceptable and feasible training period	No information of relevance was reported	No information of relevance was reported
**Watson & Stewart 2011**	Political	Focus groups were convened before (and after) the study to 1) explore pharmacists' perceptions of barriers and facilitators to delivering the intervention 2) explore with members of the public their opinions/beliefs about the intervention in community pharmacy setting	No information of relevance was reported	No information of relevance was reported
**Smoking cessation**				
**Bauld 2011**	Political, Economic	The study authors acknowledge the assistance provided by NHS Greater Glasgow and Clyde staff and the study steering group, but it is not clear where (or how) they were involved during planning	The study authors acknowledge the assistance provided by NHS Greater Glasgow and Clyde staff and the study steering group, but it is not clear where (or how) they were involved during delivery	The authors do not discuss the sustainability of the Pharmacy-led intervention, but they do conclude that it is appropriate that different cost-effective service configurations, such as pharmacy services, are available and can coexist to offer smokers choice and maximise accessibility
**Bock 2010**	Political	No information of relevance was reported	No information of relevance was reported	No information of relevance was reported
**Burford 2013**	Economic	No information of relevance was reported	No information of relevance was reported	No information of relevance was reported
**Costello 2011**	Political	This study was nested within a larger host study Smoking Treatment for Ontario Patients	During intervention delivery, the STOP Program collaborated with different community and regional	Authors highlight that reimbursement is needed to the pharmacist for providing the service

Study	Implementation context[1]	Consultation/ collaboration processes during planning[2]	Consultation/ collaboration processes during deliver[3]	Sustainability[4] ***(Note: In most studies, where relevant, pharmacists received reimbursement for providing the intervention)***
		(STOP). During planning, the STOP Program collaborated with different community and regional partners in many different ways including: tertiary-care centres; public health units; mass distribution; community pharmacies; community health centres; STOP on the road workshops with primary health units, internet-based enrolment; family health teams; and family physicians	partners in many different ways including: tertiary-care centres; public health units; mass distribution; community pharmacies; community health centres; STOP on the road workshops with primary health units, internet-based enrolment; family health teams; and family physicians	in order for it to be sustainable (There was no financial reimbursement for the pharmacists' professional services in this study). They also state that a secondary aim of the study was sustainability through training of pharmacists to provide counselling.<br>The authors state that they look forward to maintaining their existing partnerships as well as building new community connections into the future
**Crealey 1998**	Economic	No information of relevance was reported	No information of relevance was reported	No information of relevance was reported
**Hoving 2010**	No specific implementation context (study focus was simply on testing the intervention)	The intervention was developed by the University of Maastricht in collaboration with the Dutch Foundation on Smoking and Health (Stivoro for a smoke free future)	No information of relevance was reported	No information of relevance was reported
**Howard-Pitney 1999**	No specific implementation context (study focus was simply on testing the intervention)	The study authors acknowledge the assistance provided by the Shasta County Department of Public Health and Tehama County Health Agency, but it is not clear where (or how) they were involved during planning	The study authors acknowledge the assistance provided by the Shasta County Department of Public Health and Tehama County Health Agency, but it is not clear where (or how) they were involved during delivery	No information of relevance was reported.
**Maguire 2001**	Political	No information of relevance was reported	No information of relevance was reported	Barriers emerging from the qualitative evaluation of this intervention included insufficient remuneration for pharmacists which would impact on sustainability

Study	Implementation context[1]	Consultation/ collaboration processes during planning[2]	Consultation/ collaboration processes during deliver[3]	Sustainability[4] ***(Note: In most studies, where relevant, pharmacists received reimbursement for providing the intervention)***
**Mochizuki 2004**	No specific implementation context mentioned in the abstract (English abstract only)	No information of relevance was reported in the abstract	No information of relevance was reported in the abstract	No information of relevance was reported in the abstract
**Sinclair 1998**	Political, Economic	No information of relevance was reported	No information of relevance was reported	No information of relevance was reported
**Sonderskov 1997**	Political	The pharmaceutical company (Ciba–Geigy) provided instructions concerning trial procedure during planning	The pharmaceutical company (Ciba–Geigy) were in contact with pharmacies once a week during delivery	No information of relevance was reported
**Vial 2002**	Political	No information of relevance was reported	No information of relevance was reported	No information of relevance was reported
**Weight management**				
**Ahrens 2003**	Political	No information of relevance was reported	No information of relevance was reported	No information of relevance was reported
**Bush 2011**	Political	No information of relevance was reported	No information of relevance was reported	No information of relevance was reported
**Jolly 2011**	Political	No information of relevance for the pharmacy-led intervention was reported	No information of relevance for the pharmacy-led intervention was reported	No information of relevance for the pharmacy-led intervention was reported
**Malone 2003**	Political	No information of relevance was reported	No information of relevance was reported	No information of relevance was reported
**Phimarn 2013**	Political	The intervention (in Thailand) required a formal agreement between a pharmacy and a primary care unit	No information of relevance was reported	No information of relevance was reported

Note: In the majority of cases, the information in this Table was difficult to extract from the included studies, and we are cognisant that a degree of interpretation was required. The information in this Table was extracted by one reviewer (Summerbell) and checked by a second (Brown).

1. Does the study provide any useful contextual information relevant to the implementation of the intervention (e.g. political, economic, social or managerial factors)? *Note: Information in the introduction/background sections to included papers was most likely to inform the implementation context.*

Political: the primary purpose for developing and testing the intervention was the national political drive to extend the public health role of community pharmacies.

Economic: the primary purpose for developing and testing the intervention was to assess whether existing services could be delivered at a lower cost in pharmacies (and usually by pharmacists and pharmacy staff) compared with other settings and service providers.

Social: the primary purpose for developing and testing the intervention was to assess the reach of services to those most in need in pharmacies compared with similar services in other settings and service providers.

Managerial: the primary purpose for developing and testing the intervention was to assess whether existing services set in pharmacies and delivered by pharmacists could be delivered equally effectively by pharmacy assistants

2. Is there a report of consultation/collaboration processes between managers, employees and any other relevant stakeholders during the planning of stage?

3. Is there a report of consultation/collaboration processes between managers, employees and any other relevant stakeholders during the delivery of stage?

4. What is the sustainability of the intervention? – strength of the institution implementing the intervention; integration of activities into existing programmes/services/curriculum/etc.; training/capacity-building component; community involvement/participation

Supplementary file 5: Organisation and delivery of interventions

Author	Type and location of Pharmacy	Staff training and quality assurance.	Experience of intervention team	Resources and other intervention-related costs *(note: sources of funding appear in Supplementary file 2 and cost-effectiveness analyses are in Supplementary file 8).*
**Alcohol reduction**				
Dhital (2015)	Community Pharmacies in the London Borough of Hammersmith and Fulham	All trial pharmacists had been trained over 3.5 hours (by lead author) to deliver the intervention protocol including flexible use of the discussion topics in ways influenced by the counselling approach of motivational interviewing. In such a brief training workshop it was not feasible to aim to train the pharmacists in motivational interviewing as this approach requires ongoing supervision of practice.<br>*Quality assurance:* A two hour evening follow-up training session was arranged seven weeks after the start of the trial to address challenges and share learning across the group and was attended by 10 pharmacists.	Pharmacists and pharmacy support staff	Not reported
Watson & Stewart (2011)	Community Pharmacies in Grampian, Scotland	Two training sessions were delivered. One evening training session was delivered to describe the purpose of the study, the use of FAST (Fast Alcohol Screening Tool), and the study documentation. One pharmacist and up to one member of staff from each pharmacy were invited to attend. Pharmacies not represented at this event received a training visit from a research team member. A one-day ABI (Alcohol Brief Intervention) training session was also delivered to pharmacists in the intervention group, attendance at which was compulsory for participation in the study. This training was provided by Create Consultancy and the research team.<br>*Quality assurance:* none reported	Pharmacists and pharmacy support staff	Estimated cost for delivering one ABI was £70.90, based on an average of 10 people screened for each ABI delivered: £10.20 training costs, £50.00 staff time for screening, £10 staff time for delivering ABI, £0.70 for consumables.
**Smoking Cessation**				
Bauld (2011)	Community pharmacies (90% in Glasgow Health Board area)	Training of pharmacists varied from attending a Glasgow Health Board or online course, to observing sessions in the pharmacy.	Pharmacists and pharmacy support staff	In comparison with a "self-quit" attempt, the pharmacy service costs an additional £7,800 per 52-week quitter, while the group service costs an

Author	Type and location of Pharmacy	Staff training and quality assurance.	Experience of intervention team	Resources and other intervention-related costs ***(note: sources of funding appear in Supplementary file 2 and cost-effectiveness analyses are in Supplementary file 8).***
		Quality assurance: none reported		additional £9,200.
Bock (2010)	Pharmacies located within large urban community health centres in US.	A 3-hr training session for the pharmacists was conducted. Pharmacists were trained using the Rx for Change tobacco cessation program (http://rxforchange.ucsf.edu; Corelli et al., 2005), which focuses on fostering self-efficacy for counselling and includes role-playing and a hands-on workshop with the various Food and Drug Administration–approved medications for smoking cessation. All counselling approaches were aligned with the 5 A's framework (ask, assess, advise, assist, arrange follow-up) as described in the Clinical Practice Guideline (Fiore et al., 2008). The pharmacists were trained to assess readiness to quit, to focus their counselling on motivational issues for those not ready to quit, and, for those ready to quit, to offer practical advice regarding quitting, discuss the importance of obtaining social support, and evaluate the appropriateness of quit smoking medications and make recommendations (the primary difference between EQ and EQ+ conditions being the availability of free nicotine replacement therapy [NRT]). Additionally, the training addressed (a) study aims and the research protocol, (b) a demonstration of the EQ program and examination of tailored intervention reports for the patient and pharmacist, and (c) role-playing with case scenarios that integrated output from the EQ system.<br>*Quality assurance:* none reported	Pharmacists	No information provided except for incentivisation. All participants were compensated $20 for their time and effort for completing the baseline survey and for returning the follow-up survey.
Burford (2013)	Community pharmacies located around Perth city centre in Western Australia	No training details provided.<br>Unclear whether it was the community pharmacists (who would have needed some training in the use of the Face Aging software) or a single research pharmacist.	Unclear whether it was the community pharmacists or a single research pharmacist	The Face Aging software (APRIL) was provided by the software company. Total costs of implementing the intervention from a health sector perspective were

Author	Type and location of Pharmacy	Staff training and quality assurance.	Experience of intervention team	Resources and other intervention-related costs ***(note: sources of funding appear in Supplementary file 2 and cost-effectiveness analyses are in Supplementary file 8).***
		Quality assurance: none reported		AU $463, or the equivalent of AU $5.79 per participant.
Costello (2011)	Pharmacies in Ontario, Canada	Pharmacists were trained in the intervention methodology during a 5-hour face-to-face session or a 3-hour pre-recorded online session plus 1-hour teleconference conducted by the STOP Study staff. Training covered: (1) the study protocol and documentation; (2) the ‘‘5-A’’ model for brief behavioural counselling; and (3) an overview of NRT products and their use.<br>*Quality assurance:* none reported	Community pharmacists	Resources listed (but not costed) included free NRT, training, and pharmacists time.
Crealey (1998)	Belfast pharmacies	Each study site pharmacist was sent a copy of the PAS model documentation, together with a written literature review on smoking cessation and asked to study the material. Two to 3 weeks after receipt of the documentation, pharmacists attended a local workshop on smoking cessation (including detailed instruction on the study methodology).These workshops each lasted 3 hours and covered epidemiology, smoking statistics, the use of NRT, the cycle of change model and the PAS model. *Quality assurance* Following the training, a researcher visited the pharmacists to provide support and to address any queries they had in implementing the model. This constituted the training for the intervention.<br><br>*Quality assurance:* none reported	Community pharmacists	Fixed costs of the intervention are detailed in Table II. Variable costs included pharmacist time - an average time of 1 hour (over the 6-month follow-up period) at £30 per hour.
Hoving (2010)	Pharmacies in the Netherlands	Note: Training for pharmacy staff not relevant for this intervention. Computer-tailored letter (intervention) or a thank you letter (control).<br><br>*Quality assurance:* not relevant	Not relevant	Not reported.
Howard-Pitney 1999	Pharmacies in the US	Pharmacists were trained initially during a 4-hr training session with investigators and field staff. Training included educating the	Pharmacists	Not reported, but NRT was offered free of

Author	Type and location of Pharmacy	Staff training and quality assurance.	Experience of intervention team	Resources and other intervention-related costs *(note: sources of funding appear in Supplementary file 2 and cost-effectiveness analyses are in Supplementary file 8).*
		pharmacists about chewing tobacco prevalence in their counties, study protocol, nicotine withdrawal symptoms, and role of nicotine patches in reducing physical withdrawal. In addition, field staff demonstrated the pharmacists' role in the intervention protocol, and each pharmacist practiced the intervention in a role-playing exercise.<br>*Quality assurance:* At the end of the training session, field staff, playing the role of a study participant, tested the pharmacists' knowledge and ability to perform their intervention role. Pharmacists had to perform 80% of the steps adequately in each visit's protocol before being certified to intervene with participants		charge.
Maguire 2001	Pharmacies in London and Northern Ireland	Each study site pharmacist was sent a copy of the PAS model documentation, together with a written literature review on smoking cessation and asked to study the material. Two to 3 weeks after receipt of the documentation, pharmacists attended a local workshop on smoking cessation (including detailed instruction on the study methodology).These workshops each lasted 3 hours and covered epidemiology, smoking statistics, the use of NRT, the cycle of change model and the PAS model.<br>*Quality assurance:* Following the training, a researcher visited the pharmacists to provide support and to address any queries they had in implementing the model. This constituted the training for the intervention.	Community pharmacists	Not reported but authors refer to their earlier paper (Crealey et al, 1998) which does report on costs and cost effectiveness of PAS model
Mochizuki 2004	Pharmacies in Japan	Note: This paper is written in Japanese and we only have the abstract in English. There is no mention in abstract of pharmacists receiving training.	Assume pharmacists	Not reported
Sinclair 1998	Non-city community pharmacies in Grampian, Scotland	Delivery of a 2-hour training session to pharmacists and pharmacy assistants. The training did not include motivational interviewing techniques to encourage smokers to move from pre-contemplation to contemplation; however, it did include specific content and recommendations pertaining to preparation, action, maintenance, and	Pharmacists and pharmacy suport staff	The overall cost of the intervention was £14,915.76 and control costs were £14,121.13. The cost of producing one

Author	Type and location of Pharmacy	Staff training and quality assurance.	Experience of intervention team	Resources and other intervention-related costs ***(note: sources of funding appear in Supplementary file 2 and cost-effectiveness analyses are in Supplementary file 8).***
		relapse. The training aimed to give participants an understanding of the stages in the stage-of-change model, and focussed on brief questioning which could enable counsellors to assess the stage of individual customers and to subsequently increase the frequency and effectiveness of the counselling support by tailoring their advice to the current stage of the customer.<br>*Quality assurance: not reported*		additional successful attempt to quit smoking by using intensive rather than standard pharmaceutical support was £300 (in 1995-1997).
Sonderskov 1997	Community pharmacies in the areas of Aarhus and Copenhagen, Denmark	No training of pharmacists as such, but they were given "instructions" from the pharmaceutical company concerning trial procedures.<br>*Quality assurance:* pharmaceutical company contacted pharmacies at least once per week throughout the study period	Pharmacists and pharmacy support staff	Not reported, but nicotine patches were provided free of charge.
Vial 2002	Community pharmacies in Adelaide, Australia	Before the study commenced, participating community pharmacies were informed of all study-related procedures at a seminar. Brief information about stages of behaviour change and recommended interventions during smoking cessation were also included in the seminar.<br>*Quality assurance: not reported*	MSc student in health science	Not reported but nicotine patches were supplied at a half the retail cost.
**Weight management**				
Ahrens 2003	A community pharmacy – Travis Pharmacy in Shenandoah, Iowa	The two pharmacists who participated in the study received no special training, although both used current literature and research to prepare themselves to be able to counsel patients in dietary advice. A registered dietitian reviewed the dietary plan developed by the pharmacist before it was used with the patients, and was consulted as needed during the study.<br>*Quality assurance: not reported*	Community pharmacists	Not reported but meal replacements were provided free of charge.
Bush 2011	Community pharmacies in	Pharmacists did not received training, but intervention deliverers (in pharmacies and GP practices) were 'trained healthcare workers', for	A trained healthcare worker	£126.90/participant in

Author	Type and location of Pharmacy	Staff training and quality assurance.	Experience of intervention team	Resources and other intervention-related costs *(note: sources of funding appear in Supplementary file 2 and cost-effectiveness analyses are in Supplementary file 8).*
	Birmingham	example a pharmacy assistant working in a pharmacy, and they did receive training. *All intervention deliverers attended a two-day training session organised by the PCT which provided deliverers with training material and resources (p67)… The training included input from* dietitians, GP and Pharmacy staff.<br>All deliverers attended a two-day training session which was regarded as being useful and provided deliverers with training material and resources.<br><br>*Quality assurance: not reported*	(for example, a healthcare assistant, practice nurse or pharmacy assistant)	pharmacy intervention vs £100.60/participant in GP, £19.80 per participant per session in pharmacy, £20.30 per participant per session in GP
Jolly 2011	Community pharmacies, England	There are 8 arms (6 interventions) in this (Lighten Up Trial).<br>1. WW (Weight Watchers group)<br>2. SW (Slimming World, group)<br>3. RC (Rosemary Conley, group)<br>4. NHS Size Down programme (led by food advisers recruited from the local community, and trained by dietitians, group)<br>5. GP practice (nurse-led, one to one)<br>6. Pharmacy (pharmacist-led, one to one)<br>7. Control (12 free vouchers for local leisure centre)<br>8. Or a choice of one of the above.<br>1-3: the group leaders were trained by the respective organisations<br>4-6: Staff delivering these programmes had attended a three day training course on weight management in adults delivered by dietitians experienced in the management of obesity. This included key messages on diet and physical activity, doing a behavioural assessment, goal setting, plans for change, dealing with resistance, enhancing motivation, and weight maintenance. It included both practical tasks and informational components.<br>*Quality assurance: not reported*	Variable depending on intervention.<br><br>For the pharmacy-led intervention, pharmacists delivered the intervention.	Resources and other intervention costs varied between the different weight loss interventions. Interventions 4-6 (primary care) were more costly than interventions 1-3 (commercial).<br>Provider costs:<br>WW=£55.0<br>SW= £49.50<br>RC=£55.0<br>NHS SD=£70.0<br>GP=£90.86<br>Pharmacy=£90.43<br>Cost per participant (in addition to provider costs) = £10 for call centre, £3.54 for practices to

Author	Type and location of Pharmacy	Staff training and quality assurance.	Experience of intervention team	Resources and other intervention-related costs ***(note: sources of funding appear in Supplementary file 2 and cost-effectiveness analyses are in Supplementary file 8).***
				search their lists and GPs to screen lists, £8.33 for invitation letters sent by practices (£1 per letter with 12% response rate).
Malone 2003	Community pharmacies, US	Pharmacists delivering the intervention were trained (a 1 day course) in "obesity management skills". Training included various aspects of obesity but no mention of any behavioural support/skill training.<br>*Quality assurance: not reported*	Community pharmacist	Not reported
Phimarn 2013	Community pharmacy, Thailand	The two community pharmacists who provide weight loss advice routinely received minimal training. The pharmacists developed the weight loss handbook. Information included was the same as the group advice provided by the primary care unit staff. The handbook is comprised of three parts; (a) an informational section which deals with healthy diet, principles of calorie intake, food groups, portion size, and exercise, (b) a patient profile to record personal information and clinical outcomes, (c) a daily food record for patients to record their daily meals. Prior to the study, the handbook was provided to the two pharmacists as a standard guide for their use in counselling. Both community pharmacists practiced giving advice with simulated patients.<br>*Quality assurance: not reported*	Community pharmacists	Not reported

Supplementary file 6: Behaviour change

Study	Behaviour change strategy used, and theoretical basis, of intervention delivered in a community pharmacy setting, where reported.<br>***Plus same information reported for the comparison group(s) (active and/or non-active control).***	Behaviour change wheel		Nuffield intervention ladder code
		**Intervention function**	**Policy category**	
**Alcohol reduction**				
**Dhital 2015**	Brief Alcohol Intervention that was not motivational interviewing, but rather followed a structured protocol influenced by the motivational interviewing approach delivered in a 10 minute discussion; included reflection and encouraged self-directed behaviour change; feedback of the AUDIT score was also given.<br>*Comparison group: a leaflet-only control*	Education, Enablement	Service provision	Enable choice
**Watson & Stewart 2011**	Brief Alcohol Intervention based on motivational interviewing<br>*Comparison group: a general lifestyle leaflet control*	Enablement, Education	Service provision	Enable choice
**Smoking cessation**				
**Bauld 2011**	12 weeks of medium intensity behavioural counselling<br>*Comparison group: Maudsley hospital model of 7 weeks of intense group based behavioural support (not delivered in a pharmacy setting, and delivered by an 'advisor').*	Education, Enablement	Service provision	Enable choice
**Bock 2010**	All counselling approaches are aligned with the 5 A's framework (ask, assess, advise, assist, arrange follow-up) which includes counselling on motivational issues. Pharmacists deliver counselling, supported by Exper-Quit (EQ). EQ is a software system that provides individually tailored feedback to patients who smoke cigarettes, and matches reports for the pharmacist to help guide cessation counselling. Contents of the tailored feedback address the domains of motivation, decisional making (pros and cons of quitting smoking) and perceived barriers to quitting, smoking triggers/cues, nicotine dependence and effective smoking cessation medications. The tailored feedback also addresses the relationship between quitting smoking and the experience of potential negative affect and/or depressive symptoms.<br>*Comparison group: Two intervention groups included the same counselling approaches (EQ); difference was +/- free nicotine patches. The observation-only control group included no counselling.*	For either intervention groups vs control: Education, Enablement	Service provision	For EQ vs control: Enable choice<br><br>For EQ + patches vs EQ or control: Guide choice – incentives *(nicotine patches were provided free as part of the intervention)*
**Burford 2013**	The intervention group participants were digitally photoaged by using the Internet-based APRIL Face Aging software so they could preview	Education, Enablement,	Service provision	Enable choice

Study	Behaviour change strategy used, and theoretical basis, of intervention delivered in a community pharmacy setting, where reported.<br>***Plus same information reported for the comparison group(s) (active and/or non-active control).***	Behaviour change wheel		Nuffield intervention ladder code
		**Intervention function**	**Policy category**	
	images of themselves as a lifelong smoker and as a non-smoker.<br>*Comparison group: Both the intervention and control groups received '*standardised smoking cessation advice' from the pharmacist.	Persuasion		
**Costello 2011**	Comparison of two interventions that used the same behavioural counselling strategy. The pharmacists used the '5-A' model for brief behavioural counselling (see Bock, above).<br>*Comparison group: One intervention used one session, and the other used three sessions, of the behavioural counselling*	Both intervention groups:<br>Education, Enablement, Incentivisation	Service provision	Both intervention groups:<br>Guide choice – incentives *(NRT was free as part of the intervention)*
**Crealey (1998)**	Pharmacists' Action on Smoking (PAS) is a structured intervention package based on the stages of change model and using motivational interviewing. It is designed to assist smokers to stop and to motivate and support them to stay stopped, delivered in a one-to-one counselling format with structured follow up (a pilot study for the Maguire study listed below).<br>Theoretical Model: Transtheoretical model (Stages of Change)<br>*Comparison group: matched controls who did not receive PAS*	Education, Enablement,	Service provision	Enable choice
**Hoving 2010**	Computer-generated advice in *a five to seven page coherent letter* individually tailored, based on responses to a baseline questionnaire. Messages were selected through a theory-based algorithm to address aspects relevant to the individual participant (e.g. *perceived advantages and disadvantages of smoking cessation and anticipated difficult situations to refrain from smoking.*<br>Theoretical Model: I change model incorporating several cognitive models such as the transtheoretical model and theory of reasoned action.<br>*Comparison groups: a thank you letter from a pharmacist; Computer-generated advice in a letter from a GP that was individually tailored letter; a thank you letter from a GP*	Education, Enablement	Service provision Communication/ marketing	Enable choice
**Howard-Pitney 1999**	Behavioural treatment comprising of two visits to the pharmacy, support calls, and self-help materials, including a 23-page, self-help quitting manual tailored for chewing tobacco users. The major sections in the manual took the chewer through typical stages in the quitting process:	Both intervention groups:<br>Education,	Service provision, Communication/ marketing	Both intervention groups (GP and PH):<br>Guide choice –

Study	Behaviour change strategy used, and theoretical basis, of intervention delivered in a community pharmacy setting, where reported.<br>***Plus same information reported for the comparison group(s) (active and/or non-active control).***	Behaviour change wheel		Nuffield intervention ladder code
		**Intervention function**	**Policy category**	
	getting ready, quit date, dealing with urges, and recovery or staying off chew. *(Note: it was unclear whether this intervention was based on the Transtheoretical (stages of change) model)*<br>*Comparison group: same behavioural treatment as intervention, but they received a placebo patch rather than a nicotine patch*	Enablement, Incentivisation		incentives *(NRT was free as part of the intervention)*
**Maguire 2001**	Pharmacists' Action on Smoking (PAS) is a structured intervention package based on the stages of change model and using motivational interviewing. It is designed to assist smokers to stop and to motivate and support them to stay stopped, delivered in a one-to-one counselling format with structured follow.<br>Theoretical Model: Transtheoretical (Stages of change) Model<br>*Comparison group: matched controls who did not receive PAS*	Education, Enablement,	Communication/marketing, Service provision	Enable choice
**Mochizuki 2004**	Structured support from the pharmacist (5 times over 3 months)<br>*Comparison group: ad hoc advice when asked for it by participant.*	Education, Enablement	Service provision	Enable choice
**Sinclair 1998**	Behavioural counselling in smoking cessation based on the stage-of-change model. The intervention group also received nicotine patches.<br>Theoretical Model: Transtheoretical (stages of change) model<br>*Comparison groups: same as intervention but, after an initial consultation with a community pharmacist they are followed up by a research pharmacist in a hospital outpatient clinic; advice from pharmacists who have not undergone training in behavioural counselling.*	Education, Enablement	Service provision	Enable choice
**Sonderskov 1997**	The intervention did not include any behavioural support. The intervention was nicotine patches.<br>*Comparison group: same as intervention but placebo patches*	Enablement	Service provision	Guide choice – incentives (nicotine patches providedfree of charge)
**Vial 2002**	Behavioural counselling *in smoking cessation based on the stage-of-change model.*<br>*Theoretical Model: Transtheoretical (stages of change) model*<br>*Comparison groups: minimal intervention group who were provided with written material and advice only*	Education, Enablement	Service provision	Guide choice – incentives (nicotine patches were provided at about half the retail cost)

Study	Behaviour change strategy used, and theoretical basis, of intervention delivered in a community pharmacy setting, where reported. ***Plus same information reported for the comparison group(s) (active and/or non-active control).***	Behaviour change wheel		Nuffield intervention ladder code
		**Intervention function**	**Policy category**	
**Weight management**				
**Ahrens 2003**	Meal replacements<br>*Comparison group: normal low calorie diet*	Education, Enablement, Restriction	Service provision, Communication/marketing	Restrict choice
**Bush 2011**	*My Choice Weight Management Programme* delivered 'through' pharmacies. Based on the model used for the Counterweight Project with weekly consultations. Compared to the Counterweight project, there was more focus on goal setting and the targets and less focus on portion control.<br>*Comparison group: same as intervention but delivered 'through' GP surgeries*	Education, Enablement	Service provision Communication/marketing	Enable choice
**Jolly 2011**	There were 8 arms (6 interventions) in this (Lighten Up) trial. Pharmacy (pharmacist-led, one to one) was classed as the intervention group for this systematic review. The theoretical basis of the intervention was the stages of change model with use of motivational interviewing. Predominant behaviour change strategies included goal setting, self-monitoring with food diaries, hunger scale, waist measurements, and physical activity. Participants were encouraged to reward themselves for success.<br>*Comparison groups:*<br>1. *WW (Weight Watchers group) Predominant strategies used to change behaviour included stages of change, food and activity diaries, goal setting, and evaluation of progress. Rewards are given for every 3.2 kg (7 lb) lost and for loss of 5% and 10% of body weight.*<br>2. SW (Slimming World, group) Predominant behaviour change strategies used included motivational interviewing, weekly weighing; group support; and group praise for weight loss, new decisions, and continued commitment even in the absence of weight loss. Awards are given for 3.2 kg (7 lbs) lost and loss of 10% of body weight. Individual support, if needed, uses self-monitoring of food and emotions, for and against evaluations, visualisation techniques, and personal eating plans. Theoretical	Control: Enablement<br><br>Dietetic, GP and Pharmacy: Education, Enablement.<br><br>WW, SW and RC: Education, Enablement, Modelling, incentivisation.	Service provision	Dietetic, GP, Pharmacy, control: Enable choice<br><br>WW, SW and RC: Guide choice - incentives

Study	Behaviour change strategy used, and theoretical basis, of intervention delivered in a community pharmacy setting, where reported.<br>***Plus same information reported for the comparison group(s) (active and/or non-active control).***	Behaviour change wheel		Nuffield intervention ladder code
		**Intervention function**	**Policy category**	
	Model: Transactional analysis,, awareness ego states<br>3. *RC (Rosemary Conley, group) The approach is based on role modelling and group support and uses visualisation and reframing to support behavioural change. Predominant behaviour change strategies used include rewards for slimmers who maintain or lose weight, slimmer of the week, and certificates for 3.2 kg and 6.35 kg milestones. Theoretical Model: - Not reported*<br>4. NHS Size Down programme (led by food advisers recruited from the local community, and trained by dietitians, group). The theoretical background was based on the stages of change model. The benefits of physical activity, setting goals, and finding activities to fit into life were discussed. Predominant behaviour change strategies used included goal setting, stages of change, and self monitoring with a food diary. Theoretical Model: Transtheoretical (stages of change) Model<br>5. *GP practice (nurse-led, one to one) – same as intervention but different setting*<br>6. Control (12 free vouchers for local leisure centre)<br>7. *Or a choice of one of the above.*			
**Malone 2003**	Pharmacists delivering obesity management (following a training course) to patients prescribed for Orlistat.<br>*Comparison group: Orlistat plus usual care delivered by the pharmacist (who had not undertaken the training course)*	Education *(but not enablement because no behavioural component to sessions)*	Service provision	Provide information *(rather than enable choice, because no behavioural component to sessions)*
**Phimarn 2013**	Obesity counselling based on a obesity handbook *comprising of three parts; (a) an informational section which deals with healthy diet, principles of calorie intake, food groups, portion size, and exercise, (b) a patient profile to record personal information and clinical outcomes, (c) a daily food record for patients to record their daily meals.*<br>Theoretical Model: Theory of Planned Behaviour<br>*Comparison group: a routine group-directed weight management service provided by staff in the GP practice.*	Education, Enablement	Service provision	Enable choice

Supplementary file 7: Effectiveness outcomes

Study ID	Outcomes	Summary
**Alcohol reduction**		
**Dhital 2015<br>Brief alcohol intervention vs leaflet-only control**	**Alcohol Use Disorders Identification Test total scores (AUDIT) baseline:** I: 11.93 (3.24) n=205 vs. C: 11.53 (3.19) n=202<br>**12 week AUDIT:** I: 11.80 (5.88) n=168 vs. C: 10.77 (5.54) n=158<br>**12 week AUDIT change:** I:-0.11 (-0.82 to 0.61) n=168 vs. C: -0.74 (-1.47 to 0.00) n=158<br>**12 week AUDIT between group difference (95% CI) unadjusted:** -0.63 (-1.69 to 0.43)<br>**12 week AUDIT between group difference (95% CI) adjusted for pharmacist, gender, age, ethnicity, education:** -0.57 (-1.59 to 0.45)<br><br>**Other outcomes:**<br>% scoring <8 (AUDIT) at follow-up<br>3 AUDIT subscales (consumption, problems and dependence)<br>General Health Status (EQ-5D)	There was no evidence of effectiveness of community pharmacist delivery of brief alcohol intervention. The AUDIT total change score did not differ significantly between the two groups and did not change significantly between baseline and follow-up in either the intervention or control group.
**Watson & Stewart 2011<br>Brief alcohol intervention vs control**	**Fast Alcohol Screening Tool (FAST) total Median score (IQR) baseline:** I: 5.00 (3.00,6.00) n=27 vs. C: 5.00 (4.00,6.00) n=42<br>**3 month FAST Median score (IQR):** I: 3.00 (1.00,4.25) n=10 vs. C: 4.00 (2.00,6.00) n=23;<br>A reduction in FAST score of 0.93 (95% CI, -2.84 to 0.97) was shown in the intervention group at 3-months (p=0.32).<br>**6 month FAST Median score (IQR):** I: 2.50 (1.50,4.25) n=6 vs. C: 3.50 (2.00,7.50) n=14<br>**3 month FAST Mean score (sd) change:**<br>MALE: I: 0.50 (1.00) n=4 vs. C: -0.11 (3.18) n=9<br>FEMALE: I: 1.67 (2.73) n=6 vs. C: 1.17 (1.90) n=12<br>**6 month FAST Mean score (sd) change:**<br>MALE I: 2.25 (3.20) n=4 vs. C: -1.25 (2.87)n=4<br>FEMALE: I: 0.50 (0.71) n=2 vs. C: 0.75 (1.67) n=8<br>**6 month FAST between group difference(95% CI):** -1.84 (-4.49, 0.82)<br>**Other outcomes:**<br>Self-reported alcohol consumption<br>Number of alcohol-free days during an average week<br>Barriers/facilitators to delivering intervention (by pharmacists)<br>Pharmacy users opinions	No significant difference was shown between FAST score for the intervention group compared with control at 3- or 6-months.<br>At 6 months there was substantially lower follow-up of intervention clients (22.2%) compared with control clients (33.3%). Only adjusted for baseline FAST ; not clear if there were baseline differences for other variables.

Study ID	Outcomes	Summary
	Staff and training costs	
**Smoking Cessation**		
**Bauld 2011**<br><br>**I:** Pharmacy-based NHS smoking cessation service (12-weeks one-to-one support) moderate intensity + NRT<br><br>**C:** group-based NHS smoking cessation service (community-based 7 weeks behavioural support) high intensity +NRT/medication	**Baseline no. cigarettes/day >21: I: 40.1% (396/987) vs C: 41.6% (169/406)**<br><br>**4 week CO validated quitters:**<br>I: 255/1374 vs. C: 146/411 – of 1785 that set a quit date<br>I: 255/1508 vs. C: 146/471 – of 1979 who accessed service and agreed to data usage but did not set quit date<br>**52 week CO validated quitters:**<br>I: 38/1374 vs. C: 26/411 – of 1785 that set a quit date<br>I: 38/1508 vs. C: 26/471 – of 1979 who accessed service and agreed to data usage but did not set quit date<br><br>Univariate analyses: In each service more deprived smokers (those in socioeconomic groups 5 and 6) had lower cessation rates, although the trend relating socioeconomic score to cessation rate was significant only for the pharmacy service.<br>In a multivariate model, restricted to participants (*n* = 1366) with data allowing adjustment for socio-demographic and behavioural characteristics and including interaction terms, users who accessed the group-based services (C) were almost twice as likely (odds ratio 1.980; confidence interval 1.50 to 2.62) as those who used pharmacy-based support (I) to have quit smoking at 4-week follow-up.<br>**Other outcomes:**<br>4-week CO-validated quit rates by socioeconomic group score and also by Scottish deprivation quintile<br>Cost effectiveness analysis<br>Self-reported quits<br>Use of cessation aids	Much larger sample size for pharmacy than group- based service (n=1374 vs n=411). Clients could choose service. Group participants were older and of higher SES.<br>Pharmacy-based not as effective for smoking cessation but many more smokers access the pharmacy-based service.<br>All pharmacy clients had NRT, 84% group clients had NRT/16% medication<br>This is secondary data analysis of an observational study so direct comparison between the pharmacy-based and group-based service is inappropriate.
**Bock 2010**<br><br>**I1:** smoking cessation training for pharmacists and use of a computer-driven software system, "Exper_Quit", which provided individually tailored interventions and matching	**Baseline no. cigarettes/day:** I1: 18.2 (9.1) vs I2: 17.7 (8.3) vs C: 13.8 (8.6)<br>**Baseline Fagerström:** I1: 5.3; I2: 5.1; C: 4.9<br>**7-day point prevalence abstinence at 2 months** (verified with carbon monoxide (<10 ppm)): I1: 39% (39/100); I2: 27% (27/100); C: 9% (9/99)<br>**7-day point prevalence abstinence at 6 months** (verified by saliva cotinine): I1: 28% (28/100); I2: 15% (15/100); C: 8% (8/99)<br>**6 month quit between group difference OR (95% CI):**<br>I1 vs C: 3.3 (1.9 to 5.2)<br>I2 vs C: 1.49 (1.2 to 3.6)	Control group not randomised but EQ and EQ+ groups were. There were significant baseline differences and it is not clear if these were controlled for in analyses of quit rates. Low attrition.<br>A tailored intervention combined with brief proactive counselling from pharmacist plus pharmacist training (EQ) was successful in increasing quit

Study ID	Outcomes	Summary
reports to the pharmacist to guide cessation counselling plus 8 weeks free NRT<br><br>**I2:** same as above WITHOUT NRT<br><br>**C:** observation only	I1 vs I2: 2.3 (1.5 to 3.9)<br><br>**Other outcomes:**<br>Quit attempts<br>Predictors of cessation<br>Pharmacist gender (female) was positively correlated with abstinence at 2 months but not 6 months. Only 26% of participants were counselled by a female pharmacist (similar rates for EQ and EQ+ groups). Of participants who were counselled by a female pharmacist, 77% set a target quit date compared to 58% of those counselled by males.<br><br>N: EQ+: 100; EQ: 100; C: 99	rates, with further increases among patients who also received free nicotine patches (EQ+).
**Burford 2013**<br>I: standardized smoking cessation advice + computer-generated photoaging (demonstrating the detrimental effects on facial physical appearance of smoking)<br>C: standardized smoking cessation advice only	**Baseline no. cigarettes/day >21:** I: 10% (8/80) vs C: 15% (12/80)<br>**Baseline Fagerström score:** I: 2.87, C: 2.96<br><br>**6 month CO validated quitters (95% CI):** I: 11/80 (13.8%, 7.8 to 22.9) vs C: 1/80 (1.3%, 0 to 6.7)<br>This difference between groups remained statistically significant after adjustment small differences between groups in gender and nicotine dependence.<br><br>**Other outcomes:**<br>Cost effectiveness analysis<br>Nicotine dependence<br>Progression along the Transtheoretical stages of change model<br>Quit attempts<br>Self-reported quit<br>Change in Fagerström score	Photoaging intervention was effective in stopping young people smoking compared to control.
**Costello 2011**<br><br>**I1:** 1 week then fortnightly pharmacy visit for NRT plus 3 sessions of ‘‘5-A’’ model for brief behavioral counseling<br><br>**I2:** received 5 weeks NRT at initial pharmacy visit plus 1 session of ‘‘5-A’’ model for	**Baseline Heaviness of Smoking Index score 5-6 (high):** I1: 40.7% (1459/3588) vs 12: 40.1% (1364/3399) vs C: 39.4% (1823/4630)<br><br>**Self-reported 7-day point prevalence at 5 weeks:** I1: 612/3503 (17.5%) vs I2: 604/3350 (18.0%)<br><br>**Self-reported 7-day point prevalence at 5 weeks (controlling for covariates):** OR=0.96 (95% CI: 0.86 to 1.08) n=6809<br><br>**Other analyses:**<br>Study models various confounders by abstinence and intervention group and also	Control group not randomised but intervention groups were. Control group only used in paper for baseline. Only completer analysis showed significant difference between groups. When participants assessed as assigned and with non-responders classed as still smoking there is no significant difference between intervention groups.

Study ID	Outcomes	Summary
brief behavioral counseling at initial visit<br><br>**C:** 5 weeks NRT mailed directly to participants home	controls for covariates when modelling abstinence by intervention group.<br>Age and education were significant confounders; 25-39 years and 55+ were more likely to be abstinent than 18-24 years, those completing some college/university were more likely to be abstinent than those who did not complete high school.<br>Gender was not significant in “ITT” analyses (n=6809).<br>**Completer**<br>Multivariate analysis suggest when controlling for possible confounders and clustering across pharmacies group I1, 3 session **completers** were more likely to quit compared to group I2 (OR=1.72, 95% CI: 1.53 to 1.94).<br><br>**Other outcomes:**<br>Predictors of abstinence	
**Crealey 1998**<br><br>**I: PAS model of behavioural support, 35/52 nicotine gum**<br><br>**C1: Nicotine gum only**<br><br>**C2: Control (expressed wish to stop smoking)**	**Baseline cigarettes/day:** NR<br><br>**3 month CO verified abstinence (and stopped using nicotine gum): I: 56%; C1: 16%**<br>**6 month CO verified abstinence (and stopped using nicotine gum): I: 46%; C1: 6%; C2: 0%**<br><br>**N: I1: 52; I2:48; C: 60**<br>**Other outcomes:**<br>Cost effectiveness analysis	There was a statistically significant difference in cessation rates between intervention and control patients.
**Hoving 2010**<br><br>**I:** computer tailored smoking cessation letter distributed through community pharmacies<br><br>**C:** thank you letter only distributed through community pharmacies	**Baseline cigarettes/day:** I: 22, n=256 vs C: 21, n=289<br><br>**3 month continued abstinence (having refrained from smoking between baseline and follow-up, yes/no)**: I: 4/256 vs 3/289<br><br>**12 month continued abstinence (having refrained from smoking between baseline and follow-up, yes/no):** I: 2/256 vs 2/289<br><br>**Other outcomes:**<br>quit attempt<br>point prevalence	At 3 and at 12 months there was no significant difference between I vs C in the pharmacy sample except for quit attempts at 12 months: responders in the experimental group were more likely to have had a quit attempt than the control group (OR 1.48; CI 1.03-2.11, p <0.05) controlled for no. previous quit attempts.<br>There was a pharmacy setting and a GP setting - treated as 2 separate trials. GP sample not extracted as follow-up is at different time periods than pharmacy sample - there is an intervention and control group for both pharmacy and GP settings (4 groups).

Study ID	Outcomes	Summary
		GP & 15 pharmacies used passive recruitment, 50 pharmacies used active recruitment.
**Howard-Pitney 1999**<br><br>**I:** pharmacist advice and support + nicotine patch (free 6 week 15mg patches)<br><br>**C:** pharmacist advice and support + placebo patch	**Baseline no. cans/week:** I: 3.9 (2.4) n=206 vs C: 4.1 (2.3) n=204<br><br>**7-day point prevalence at 6 months (verified by cotinine):** I: 38% (78/206) vs C: 34% (69/204)<br><br>In intervention group age was a significant predictor of relapse (older chewers less likely to relapse).<br><br>**Other outcomes:**<br>Self-reported 7-day point prevalence at 10 days<br>Self-reported 7-day point prevalence at 3 months<br>self-reported relapse (first day chewed tobacco for 7 days in a row)<br>Predictors of relapse	Relatively old study of tobacco chewers. Abstinence rates relatively high at 6-month follow-up but not significantly different between groups.
**Maguire 2001**<br><br>**I:** Pharmacist Action on Smoking (PAS) model, 87% NRT<br><br>**C:** ad hoc pharmacist advice on smoking cessation, 84% NRT	**Baseline no. cigarettes/day**<br>**1-10:** I: 14/265; C: 26/219<br>**10-20** I: 197/265; C: 121/219<br>**20-30:** I: 29/265 vs C: 33/219<br>**>30:** I:13/265; C: 20/219<br>**12 month abstinence** (self-reported abstinence since the intervention for 12 months supported by a negative urinary cotinine test at 12 months)**:** I: 14.3% (38/265) vs C: 2.7% (6/219)<br><br>**Other outcomes:**<br>Self-reported abstinence at 3 and 6 months	The PAS intervention significantly increased smoking cessation compared with control. It is unclear how many of the participants actually reached 12 months of follow-up. Pharmacists were willing to participate before randomisation.
**Mochizuki 2004**<br><br>**I:** Nicotine gum plus advice on usage and initial and follow-up cessation advice<br><br>**C:** Nicotine gum plus advise on usage	**Baseline no. cigarettes/day:** I: 23.0 (6.75) n=11 vs C: 25.7 (13.9) n=16<br>**Baseline Fagerström: I: 4.56 (2.13) vs C 6.31 (1.85)**<br><br>**Self-reported complete cessation (no smoking and no use of nicotine gum) at 3-months:** I: 45.5% (5/11) vs C: 31.2 (5/16);<br>OR 1.83 (not statistically significant)<br><br>**Other outcomes:**<br>relationship between the smokers ergogram and effectiveness of the intervention	Both interventions appear to increase cessation but not reported if significant improvement from baseline, no significant difference between groups at follow-up. Very small study.
**Sinclair 1998**	**Baseline no. cigarettes/day: NR,**	The intervention was associated with a

Study ID	Outcomes	Summary
**I:** training pharmacists and pharmacy assistants in the stage-of-change model of smoking cessation<br><br>**C:** standard professional support	**Baseline Fagerström:** I: 5.2 n=224 C: 5.2 n=263<br><br>**Self-reported continued abstinence at 9 months:** I: 12% (26/217) vs C: 7.4% (19/257)<br>**Self-reported continued abstinence at 9 months between group difference (95% CI):** 4.6% (-0.8 to 10.0)<br>Outcome was not affected by sex, age, socioeconomic status (Carstairs Morris deprivation score (1992))<br><br>**Other outcomes:**<br>Self-reported point prevalence at 1 month<br>Self-reported continued abstinence at 4 months<br>Perceptions of customers and pharmacy personnel<br>Cost effectiveness analysis	favourable non-significant trend at 9 months based on self-reported abstinence. Pharmacists were willing to participate before randomisation. The study failed to reach its recruitment target (about half); power was reduced to the 10% level.
**Sonderskov 1997**<br><br>**I1:** free 21 mg nicotine patches (12 weeks, dosage reduced)<br><br>**I2:** free 14 mg nicotine patches (12 weeks, dosage reduced)<br><br>**C1:** free placebo 21 mg patches (12 weeks, dosage reduced)<br><br>**C2:** free placebo 14 mg patches (12 weeks, dosage reduced)<br><br>No psychological or behavioural support was added to the pharmacologic treatment.	**Baseline no. cigarettes/day:**<br>**10-14:** I1: 2/136; I2: 51/119; C1: 0/142; C2: 53/125<br>**15-19:** I1: 9/136; I2: 62/119; C1: 12/142; C2: 64/125<br>**20-24:** I1: 88/136; I2: 3/119; C1: 92/142; C2: 5/125<br>**≥25:** I1: 37/136; I2: 0/119; C1: 38/142; C2: 0<br><br>**Self-reported point prevalence at 26 weeks (no smoking during a 4-week treatment period or one episode of a slip defined as <6 days of smoking within a 4-week period):** I1: 11% (15/132) vs C1: 4.2% (6/142); I2: 22.7% (27/119) vs C2: 18.4% (23/125)<br><br>**Prevalence proportion ratio (95% CI):** I1 vs C1: 2.61 (1.04 to 6.53)<br>**Prevalence proportion ratio (95% CI):** I2 vs C2: 1.23 (0.75 to 2.03)<br><br>**Other outcomes:**<br>Self-reported point prevalence at 4, 8 and 12 weeks	Relatively old study. Self-reported point prevalence only which also includes participants who have one episode of smoking (<6 days).<br><br>Intervention effective for those smoking ≥20/day at baseline randomised to I1 (21mg patches) vs C1, but not effective for lighter smokers randomised to 12 (14mg patches) vs C2. However it appears that both intervention and placebo 14mg patch groups had more quitters compared to 21mg patch intervention and control groups (not statistically tested). Seems to be a placebo effect especially in low dose placebo group.<br>Noncompliance among successful quitters was low.
**Vial 2002**	**Baseline no. cigarettes/day:** NR, Fagerström score mean (range): I1: 5.79 (3-9) n=34; I2: 5.94 (1-9) n=35; C: 6.33 (1-9) n=33	Participants were all former inpatients of respiratory unit and intervention

Study ID	Outcomes	Summary
**I:** community pharmacy-based nicotine patches plus weekly counselling ($15.00 weekly patches x 16 weeks)<br><br>**I2:** hospital outpatient clinic nicotine patches plus weekly counselling ($15.00 weekly patches x 16 weeks)<br><br>**C:** minimal intervention (written and verbal information at baseline)	**Self-reported continued abstinence at 12 months (not smoked since discharge) :** I1: 19% (4/21) vs I2: 24% (5/21) vs C: 4.6% (1/22)<br><br>**Other outcomes:**<br>Self-reported continuous abstinence at 3 and 6<br>Self-reported continuous abstinence x compliance<br>Self-reported 7-day point prevalence at 3 and 6 months<br>Self-reported 7-day point prevalence x compliance<br>Self-reported 30-day point prevalence at 12 months	commenced for all participants whilst inpatients then continued after discharge (either as outpatient or pharmacy-based).<br><br>Point prevalence but not continuous abstinence was significantly different in favour of either active intervention compared to control at 12 month follow-up.
**Weight management**		
**Ahrens 2003**<br><br>**I1: Meal replacement (free products)**<br><br>**I2: Conventional low-calorie diet both set in community pharmacy**	**BMI (kg/m^2): baseline:** I: 29.5 (2.2) n=45 vs. C: 29.0 (2.6) n=43<br>**BMI (kg/m^2) change: NR**<br><br>**WC (cm) baseline:** I: 89.1 (8.5) n=45 *vs.* C: 87.0 (8.2) n=43<br>**12 week WC (cm) change:** I:-5.31 n=45 *vs.* C: -6.10 n=43<br>**22 week WC (cm) change:** I:-8.08 n=45 *vs.* C: -7.82 n=43<br>**12 week WC (cm) between group difference (95% CI):** NR<br><br>**WT (kg) baseline:** I: 81.9 (11.1) n=45 *vs.* C: 78.3 (10.1) n=43<br>**WT (kg) 12 weeks:** I: 77.0 (1.6SE) n=45 *vs.* C: 74.0 (1.6SE) n=43<br>**12 week WT (kg) change:** I: −4.9 (0.3SE) n=45 vs. C:-4.3 (0.3SE) n=43<br>**12 week WT (kg) change:** I: −5.2(0.4SE) *vs.* C:-4.3 (0.4SE) N=68 (I + C)<br>**12 week WT (kg) between group difference (95% CI): -**0.9 (−2.0 to -0.1) N=68 (I + C)<br>**12-22 week WT (kg) change:** I: −0.7(0.4SE) *vs.* C:-0.9 (0.4SE) N=68 (I + C)<br>**12-22 week WT (kg) between group difference (95% CI):** 0.3 (−0.8 to -1.4) N=68 (I + C)<br><br>**Other outcomes:**<br>% WT loss<br>>7% body WT loss<br>DBP<br>SBP<br>Triglycerides	During the 12-week weight loss phase both groups lost a significant amount of WC and WT, the MR group also lost significant amount of WT between weeks 12-22, although no significant difference between the groups at 12-weeks or at 22-weeks.<br>High dropout especially during maintenance phase.

Study ID	Outcomes	Summary
	HDL-C<br>LDL-C<br>TC	
**Bush 2011**<br>**Diet and exercise with a trained healthcare worker (healthcare assistant, practice nurse, pharmacy assistant):**<br>**I: pharmacy-based**<br>**C: GP-based**	**BMI (kg/m^2) baseline:** I: 33.0 n=unclear *vs.* C: 35.6 n=unclear<br>**12-week BMI (kg/m^2) change:** I: -0.9 (0.2) n=91 *vs.* C: -1.4 (0.3) n=75<br>**15 week BMI (kg/m^2) change:** I: −1.3 (0.4) n=60 *vs.* C:-0.8 (0.7) n=22<br>**BMI (kg/m^2) between group difference (95% CI):** NR<br><br>**WC (cm) baseline:** I: 105.1 *vs.* C: 108.8<br>**12 week WC (cm) change:** I: -4.9 (0.9) n=91 *vs.* C: -6.0 (1.3) n=75<br>**15 week WC (cm) change:** I: -6.5 (1.6) n=60 *vs.* C: -4.9 (2.6) n=22<br>**WC (cm) between group difference (95% CI):** NR<br><br>**WT (kg) baseline:** I: 86.1 N=186 *vs.* C: 95.8 n=268<br>**12 week WT (kg) change: I**: −2.4(0.6) n=91 *vs.* C: -3.8 (0.8) n=75<br>**15 week WT (kg) change: I**: −3.4(1.1) n=60 *vs.* C: -2.3 (1.9) n=22<br>**WT (kg) between group difference (95% CI):** NR<br><br>**Other outcomes:**<br>12-week % WT by sex, age, indices of multiple deprivation (IMD) and ethnicity<br>Cost effectiveness analysis<br>12 week health-related quality of life (Short Form-12)	Significant differences between groups at baseline. GP participants tending to be older than their pharmacy. Greater % Asian participants recruited in pharmacies. Large dropout and small groups at last follow-up. Both groups appear to reduce BMI, WC and WT at follow-up, statistical significance either from baseline to follow-up or between groups are not reported. Pharmacy group appear to continue to improve between weeks 12 to 15 but the GP group outcomes do not.<br>There were no statistically significant relationships between sex, age, IMD quintile or ethnicity and % WT loss at session 12 within pharmacy or GP participants. Completer analysis only.<br>Attendance rates on the Programme were consistently better among pharmacy participants than among GP participants.
**Jolly 2011**<br>**Weight Watchers, Slimming World, Rosemary Conley, Size Down (NHS community-based), GP, Pharmacy, participants own choice vs control (exercise)**	**BMI (kg/m^2) baseline:** WW: 33.96 (3.9); SW: 33.83 (3.8); RC: 33.38 (3.5); SD: 33.77 (3.9); GP: 33.06 (3.5); Pharmacy: 33.44 (3.5); Choice: 33.41 (3.4); C: 33.88 (4.4)<br><br>**BOCF 12-week BMI (kg/m^2) change: NR**<br>**BOCF 1 year BMI (kg/m^2) change:** WW: -1.17 (-1.7 to -0.7); SW: -0.71 (-1.0 to -0.4); RC: -0.75 (-1.1 to -0.3); SD: -0.67 (-1.0 to -0.3); GP: -0.32 (-0.7 to 0.1); Pharmacy: -0.31 (-0.7 to 0.0); Choice: -0.90 (-1.3 to -0.5); C: -0.45 (-0.8 to -0.1)<br>**BOCF BMI (kg/m^2) between group difference (95% CI) adjusted for weight at baseline, physical activity at baseline, age, sex, and ethnic group:**<br>**WW vs C: −2.34 (−3.56 to -1.13)**<br>**SW vs C: −1.24 (−2.47 to -0.02)**<br>**RC vs C: −2.39 (−3.61 to -1.16)**	All programmes achieved significant weight loss ranging from -1.37 kg (GP) to -4.43 kg (Weight Watchers) at 12 weeks.<br>All except GP and pharmacy groups resulted in significant weight loss at one year. At one year, only the Weight Watchers group had significantly greater weight loss than did the control (exercise only) group (2.5 kgs, 95% CI 0.8 to 4.2).<br>The commercial programmes (Weight Watchers, Slimming World, and

Study ID	Outcomes	Summary
	**SD vs C: −0.09 (−1.31 to 1.14)**<br>**GP vs C: 0.61 (−0.73 to 1.96)**<br>**Pharmacy vs C: 0.12 (−1.51 to 1.27)**<br>**Choice vs C: −1.33 (−2.55 to -0.11)**<br><br>**WC (cm) baseline:** NR<br><br>**WT (kg) baseline:** I: *vs.* C:<br>**BOCF 12 week WT (kg) change:** WW: -4.43 (-5.3 to -3.6); SW: -3.56 (-4.4 to -2.7); RC: -4.23 (-5.2 to -3.2); SD: -2.38 (-3.1 to -1.7); GP: -1.37 (-2.3 to -0.4); Pharmacy: -2.11 (-3.2 to -1.0); Choice: -3.32 (-4.1 to -2.5); C: -2.01 (-2.8 to -1.2)<br>**BOCF 1 year WT (kg) change:** WW: -3.46 (-4.8 to -2.1); SW: -1.89 (-2.9 to -0.9); RC: -2.12 (-3.4 to -0.9); SD: -2.45 (-3.6 to -1.3); GP: -0.83 (-2.0 to 0.4); Pharmacy: -0.66 (-1.7 to 0.4); Choice: -2.15 (-3.4 to -0.9); C: -1.08 (-2.1 to -0.1)<br>**WT (kg) between group difference (95% CI) adjusted for weight at baseline, physical activity at baseline, age, sex, and ethnic group:**<br>**WW vs C: −2.49 (−4.15 to -0.83)**<br>**SW vs C: −0.90 (−2.57 to 0.77)**<br>**RC vs C: −1.35 (−3.03 to 0.33)**<br>**SD vs C: −1.65 (−3.33 to 0.04)**<br>**GP vs C: 0.12 (−1.96 to 1.72)**<br>**Pharmacy vs C: 0.06 (−1.84 to 1.96)**<br>**Choice vs C: −1.47 (−3.13 to 0.20)**<br><br>**Other analyses:**<br>Completers<br>LOCF<br>Unadjusted between group difference (BMI, WT)<br><br>**Other outcomes:**<br>% WT loss<br>>5% body WT loss<br>Costs<br>Physical activity<br><br>**N:** WW:100; SW: 100; RC: 100; SD: 100; GP: 70; Pharmacy: 70; Choice: 100; C: 100	Rosemary Conley) achieved significantly greater weight loss than did the primary care programmes (general practice and pharmacy based interventions) at 12 weeks and 1 year. At one year, the difference was 1.6 (0.3 to 2.9) kg (P=0.06) in the adjusted model. Mean weight loss at one year, with baseline value used for imputation was 0.8 (SD 4.7) kg for primary care and 2.5 (6.2) kg for commercial programmes.
**Malone 2003**	**BMI (kg/m^2) baseline:** I: 48.3 (14.6) *v.* C: 42.8 (8.1)	Very small study, high dropout, high

Study ID	Outcomes	Summary
 I: orlistat + usual outpatient care + community pharmacy support<br><br>**C: orlistat + usual outpatient care**	**BMI (kg/m^2) change: NR**<br><br>**WC (cm) baseline:** I: 128 (20) *vs.* C: 127 (17)<br>**WC (cm) change: NR**<br><br>**WT (kg) baseline:** I: 130 (39) *vs.* C: 124 (30)<br>**26 weeks WT (kg) change:** I: −3.5(2.9) *vs.* C: -3.0 (5.2)<br>**WT (kg) between group difference (95% CI):** NR<br><br>N: I: 15; C: 15<br><br>**Other outcomes:**<br>% WT loss<br>> 3% body WT loss<br>General health status (SF-36)	baseline BMI. Both groups appeared to lose similar amount of WT at 26 weeks.
**Phimarn 2013**<br><br>**I: Community pharmacist individual support**<br><br>**C: Primary care unit group support**	**BMI (kg/m^2) baseline:** I: 27.49 ± 3.15 *vs.* C: 27.74 ± 3.25<br>**16 weeks BMI (kg/m^2):** I: 26.68±4.88 *vs.* C: 27.93 ±3.30<br>**BMI (kg/m^2) change: I: -0.80±0.07 vs. C: 0.19 ±0.04**<br>**BMI (kg/m^2) between group difference (95% CI): NR**<br><br>**WC (inches) baseline:** I: 36.26 ± 3.50 *vs.* C: 37.23 ± 3.02<br>**16 weeks WC (inches):** I:36.30 ±3.56 *vs.* C: 37.12 ±3.01<br>**WC (inches) change:** I: 0.04 ±0.01 vs. C: -0.11 ±0.03<br>**WC (cms) change:** I: 0.1 ±0.03 vs. C: -0.28 ±0.08<br><br>**WC (cm) between group difference (95% CI):** NR<br><br>**WT (kg) baseline:** I: 66.80 ± 7.44 *vs.* C: 66.66 ± 8.03<br>**16 weeks WT (kg): I: 65.98 ±7.15 vs. C: 67.58 ±7.98**<br>**WT (kg) change:** I: -0.82 ±0.29 *vs.* C: 0.92 ±0.19<br>**WT (kg) between group difference (95% CI):** NR<br><br>**N:** I:33 *vs.* C:33<br><br>**Other outcomes:**<br>Theory of Planned Behaviour scores	Neither group showed significant improvement in clinical outcomes. Small study. Completer analysis only.

Supplementary File 8: Economic evaluations

Study characteristics	Data	Results
**Alcohol reduction**		
**Author:** Watson & Stewart 2011;<br>**Type of economic evaluation:** cost analysis<br>**Description of intervention:** screening and brief alcohol advice vs general lifestyle leaflet control	**Perspective:** health sector<br>**Data:** costs derived from financial records maintained by research team also pharmacy logs. Training costs annuitized over 3 years, staff costs based on fee payment.	**The overall cost for delivering the brief intervention was £70.90, or £6.09 per person screened,** based on an average of 10 people screened for each brief intervention delivered.<br>Alternative assumptions produce lower or higher costs - sensitive to staff time cost, number of clients screened per pharmacy and number of clients screened per brief intervention delivered. Time taken to deliver brief intervention was very variable (range: 5 to 42 minutes) with no systematic pattern.
**Smoking cessation**		
**Author:** Bauld 2011<br>**Type of economic evaluation:** cost-utility analysis<br>**Description of intervention:** individual pharmacy-based NHS smoking cessation service + NRT and group community-based NHS smoking cessation service + NRT vs self-quit scenario	**Perspective:** health sector (Glasgow NHS Health Board)<br>**Data:** study outcome data used to derive costs and probabilities of quitting of 0.025 and 0.055 for the pharmacy and group-based interventions respectively and 0.015 for self-quit - slightly more conservative estimate as used initial sample (1,508 pharmacy cases and 471 group cases) rather than quit date sample.<br>Markov model had 4 main Markov states: ex-smoker, smoker, death and smoking-related death. Risk of relapse up to 8 years post-quit; risk of smoking-related death for an ex-smoker limited to 12 years post-quit. Model lifespan 75 years. Discount rate (QALYs) 3.5%. Costs from 2007.	Services were not directly compared (different participant demographics) but were both compared to a baseline self-quit scenario.<br>The group service achieved a higher quit rate (6.3%) than the pharmacy service (2.8%) but was more intensive and required greater overhead costs. In comparison with a "self-quit" attempt, the pharmacy service costs an additional £7,800 per 52-week quitter, while the group service costs an additional £9,200.<br>**Cost effectiveness (52-week quitter) of intervention per client: One-to-one pharmacy led intervention £79.00; Group-based support £368.00.**<br>**Incremental cost per QALY of £2,600 for pharmacy one-to-one counselling and £4,800 for the group support.**
**Author:** Burford 2013<br>**Type of economic evaluation:** cost-effectiveness analysis<br>**Description of intervention:** smoking cessation advice + computer-generated photoaging vs	**Perspective:** health sector and also a community pharmacy (based on the assumption that the intervention was not government funded)<br>**Data:**<br>Direct costs: time taken to provide the service (rate	**Total costs of implementing the intervention from a health sector perspective were AU $463, or the equivalent of AU $5.79 per participant.**<br>With an additional 10 quitters confirmed in the intervention group compared to the control group

Study characteristics	Data	Results
smoking cessation advice	of pay) and the cost to a pharmacy of purchasing tokens (market price) to use the online software to photoage participants.<br>Potential cost offsets from a reduction in health care costs of quitters were used to calculate net intervention costs (Quit Benefits Model, after 10 years follow-up). Cost offsets discounted at 3%, costs in 2011 Australian dollars.<br>The number of lifetime quitters was calculated assuming a long-term smoking relapse rate of 37% within 10 years.<br>Scenario sensitivity analysis with the best-case and worst-case scenarios. Parameters varied were the pharmacist's time spent providing the service, the exchange rate for converting the cost of tokens from American dollars to Australian dollars, and the discount rate.<br>Also quantitative data from the customer survey and customers' perceptions about the value of the intervention and its impact on loyalty intentions and potential future sales.	(11 and 1, respectively), the **ICER was AU $46 per additional quitter, or the equivalent of AU $74 per additional lifetime quitter.**<br>Cost offsets of AU $2144 from a reduction in the health care costs of quitters resulted in the intervention potentially generating net total cost savings of AU $1778.<br>In the best-case scenario, the ICER was AU $41 per additional quitter and net total cost savings were AU $2346. Corresponding figures for the worst-case scenario were AU $71 per additional quitter and AU $1316, respectively.<br>**The mean cost that the participants indicated that they were willing to pay for the digital aging service was AU $20.25,** which exceeded the mean cost per participant for delivering the service (AU $5.79).
**Author:** Crealey 1998<br>**Type of economic evaluation:** cost-effectiveness analysis<br>**Description of intervention:**<br>behavioural support (formal counselling, 'Pharmacists Action on Smoking' model) 67% (35/52) nicotine gum vs nicotine gum only vs control (expressed wish to stop smoking)	**Perspective:** health sector<br>**Data:**<br>Direct costs only, expected outcomes and published data on the increase in life-years saved when a patient stops smoking.<br>Various assumptions i.e. 100% uptake by pharmacies, 20 patients recruited per pharmacy per year, 10% quit rate at 12 months, 10% relapse rate, 1% quit rate in absence of intervention, fixed costs (materials and training) of £55,000, variable costs per patient entering intervention (average 1 hour over the 6-months at cost of £30 per hour consultancy rate for a community pharmacist).<br>Costs in 1997 pounds sterling, discount rate 4%.	**Cost per life-year saved ranged from £196.76 to £351.45 for men and from £181.35 to £772.12 for women depending on age. Given the baseline assumptions and on the basis of a 45-year-old smoker, the cost per successful intervention was £509.60.**<br>The cost of the programme was sensitive to changes in the discount rate, variable costs and the success rate of the programme. From the pilot study, the success rate was estimated from the number who stopped smoking out of the number who entered stage three of the programme (decides to make an attempt to stop smoking).
**Author:** Sinclair 1998<br>**Type of economic evaluation:** cost-effectiveness analysis	**Perspective:** Societal<br>**Data:** costs (1995) borne by the health service, pharmacies and clients; opportunity costs	The overall cost of the intervention was £14,915.76 and control costs were £14,121.13. The intervention resulted in seven more quitters at

Study characteristics	Data	Results
**Description of intervention:** training pharmacists/assistants in smoking cessation behaviour change (stages of change model) + NRT vs standard professional pharmacy support + NRT	questionnaire was completed by all the participants while one participant per pharmacy completed the pharmacy expenses claim form. Costs to the NHS (training sessions and trainees' out-of-pocket expenses, including staff costs and travel). Travel time = 0.4 x wage rate. Any NRT purchased was a cost of the intervention to the client. The cost of the health promotion materials and pharmacy client documentation was borne by the research project, but would not ultimately be a cost to the NHS. Sensitivity analysis performed, no discounting rate.	a cost of £794.63. **ICER was £300 per additional quitter, or £83 per life year.** The key determinants of the ICER were the number of quitters, the costs of training and the costs of NRT.
**Weight management**		
**Author:** Bush 2011<br>**Type of economic evaluation:** cost-effectiveness analysis<br>**Description of intervention:** pharmacy-based diet + physical activity vs GP-based diet + physical activity	**Perspective:** health sector<br>**Data:**<br>Direct costs only; providers were reimbursed £300 for attending two days of training upon recruitment of 6 participants. Providers were reimbursed £30 for the initial assessment of each participant. Providers were reimbursed £10 for each consultation after the initial assessment.	Total cost for pharmacy = £23,230, for GP = £26,970; difference in cost is explained by the remuneration structure for the programme as payments were based on the number of sessions hosted (number of sessions hosted by GPs=1735; pharmacy=1447).<br>**£126.90/participant (n=183) in pharmacy intervention vs £100.60/participant (n=268) in GP, £19.80 per participant per session in pharmacy, £20.30 per participant per session in GP.** Costs per participant were higher in a pharmacy setting than a GP setting initially, but by the end of the programme (9 months) the costs were about the same because of the larger number of participants recruited by GPs (thus allowing for distribution of, for example, training costs across a larger pool of participants).<br>Among participants attending session 12, the cost per kg of weight loss was £57.00 with costs being higher among pharmacy providers (£74.80) than among GP providers (£43.40). Similarly costs per 1% of weight loss were £87.00 among pharmacy providers and £59.00 at GP providers (£74.30 combined).**At session 12 *ICER (£ per kg per***

Study characteristics	Data	Results
		***participant)* cost -£8.29 through pharmacy providers (favours GP). Conversely, at session 15, *ICER (£ per kg per participant)* would cost £2.91 through GP providers (favours Pharmacy).**<br>**It is unclear which provider type delivered the Programme more cost-effectively.**
**Author:** Jolly 2011*<br>**Type of economic evaluation:** Cost analysis<br>**Description of intervention:**<br>Pharmacy-based diet + physical activity;<br>Weight Watchers;<br>Slimming World;<br>Rosemary Conley;<br>NHS Size Down;<br>GP;<br>Participants own choice;<br>Exercise only control;	**Perspective:** health sector (primary care trust)<br>**Data:** direct costs only to the primary care trust, costs of the provider's service and the cost of the searches in general practice (£3.54 for practices to run search of their lists and for general practitioners to screen lists for ineligible participants), invitation letters (£8.33 for invitation letters sent by practices at£1 per letter, with 12% response rate), and provision of call centre support (average per person, based on the cost of staff employed over a 12 month period and the number of clients = £10 per call centre).	**Services were not directly compared; each intervention was compared to an exercise only control.**<br>**Total costs per participant:**<br>**Pharmacy-based £112.30;**<br>**Weight Watchers £76.87;**<br>**Slimming World £71.37;**<br>**Rosemary Conley £76.87;**<br>**NHS Size Down £91.87;**<br>**GP £112.73;**<br>Does not include any training costs for providers, costs calculated for a standard pool size of 70.<br>The primary care programmes were the most costly to provide.<br>In the most effective intervention, participants lost 1.3 kg/m2 on average, study authors recalculated the life table on the basis of this reduced BMI. The difference in life expectancy was about one year. If we assumed that the people randomised to this intervention continued to weigh 1.3 kg/m^2 less throughout life, then the **cost per life year saved was about £77** (€88; $122). These benefits are not discounted and make many assumptions, critical factor being duration of maintenance of weight loss.

* all intervention groups in the Jolly trial were compared to the exercise only control group (intervention groups were not directly compared; ICER: incremental cost-effectiveness ratio; NRT: nicotine replacement therapy; QALY: quality-adjusted life years;
